# Vanillyl alcohol oxidase from *Diplodia corticola*: Residues Ala420 and Glu466 allow for efficient catalysis of syringyl derivatives

**DOI:** 10.1016/j.jbc.2023.104898

**Published:** 2023-06-08

**Authors:** Daniel Eggerichs, Nils Weindorf, Maria Laura Mascotti, Natalie Welzel, Marco W. Fraaije, Dirk Tischler

**Affiliations:** 1 Department of Microbial Biotechnology, Ruhr-University Bochum, Bochum, Germany; 2 Department of Molecular Enzymology, University of Groningen, Groningen, The Netherlands; 3 Facultad de Química Bioquímica y Farmacia, IMIBIO-SL CONICET, Universidad Nacional de San Luis, San Luis, Argentina

**Keywords:** flavoprotein oxidase, lignin, ether cleavage, ancestral sequence reconstruction, enzyme engineering, biocatalysis

## Abstract

Vanillyl alcohol oxidases (VAOs) belong to the 4-phenol oxidases family and are found predominantly in lignin-degrading ascomycetes. Systematical investigation of the enzyme family at the sequence level resulted in discovery and characterization of the second recombinantly produced VAO member, *Dc*VAO, from *Diplodia corticola*. Remarkably high activities for 2,6-substituted substrates like 4-allyl-2,6-dimethoxy-phenol (3.5 ± 0.02 U mg^−1^) or 4-(hydroxymethyl)-2,6-dimethoxyphenol (6.3 ± 0.5 U mg^−1^) were observed, which could be attributed to a Phe to Ala exchange in the catalytic center. In order to rationalize this rare substrate preference among VAOs, we resurrected and characterized three ancestral enzymes and performed mutagenesis analyses. The results indicate that a Cys/Glu exchange was required to retain activity for ɣ-hydroxylations and shifted the acceptance towards benzyl ethers (up to 4.0 ± 0.1 U mg^−1^). Our findings contribute to the understanding of the functionality of VAO enzyme group, and with *Dc*VAO, we add a new enzyme to the repertoire of ether cleaving biocatalysts.

Vanillyl alcohol oxidases (VAOs) are a group of the 4-phenol oxidases family, which occur exclusively in ascomycetous fungi. These enzymes are hypothesized to be involved in the degradation of aromatic compounds from natural sources like lignin breakdown or amino acid transformation as the group of *vao* gene-containing ascomycetes prefers a saprophytic lifestyle or are found to be plant-pathogens and endophytes (1, 2, 3, 4). Phylogenetically, fungal VAOs are related to bacterial eugenol oxidases (EUGOs) and 4-ethyl phenol oxidases (4EPOs) (2, 5). Until now, these three groups are characterized solely by a single member each where *Ps*VAO from *Penicillium simplicissimum*, *Rj*EUGO from *Rhodococcus jostii* RHA1, and *Gc*4EPO from *Gulosibacter chungangensis* represent the VAO, EUGO, and 4EPO groups, respectively (5, 6, 7). The putative VAO from *Byssochlamys fulva* V107 has been described as well; however, no sequence information is available for this enzyme (4).

Along with alcohol oxidation, dehydrogenation, hydroxylation, and deamination reactions, *Ps*VAO also performs the cleavage of benzyl ethers which are indeed the proposed natural substrates from lignin degradation, as the expression of the corresponding encoding gene was shown to be induced by 4-hydroxy benzyl methyl ether (1, 8). This hypothesis is corroborated by the subcellular localization of *Ps*VAO in the peroxisomes through its C-terminal peroxisomal targeting signal (PTS1), as these organelles are involved in the oxidative metabolism (9). *Rj*EUGO and *Gc*4EPO share similar reaction profiles, but substantial activity on ether substrates has been reported only for *Ps*VAO so far (Fig. 1) (5, 10). This high substrate promiscuity made *Ps*VAO an interesting candidate for biocatalytic applications like the production of vanillin from capsaicin or 2-methoxy-4-methylphenol and the stereoselective hydroxylation of 4-alkylphenol derivatives (11, 12, 13, 14). Based on detailed mechanistic studies and resolved crystal structures, some crucial active site residues have been identified (15, 16). This allowed engineering of mutant enzymes displaying for example an inversion of the enantioselectivity for the hydroxylation of ethyl phenol (17, 18).

**Figure 1:**
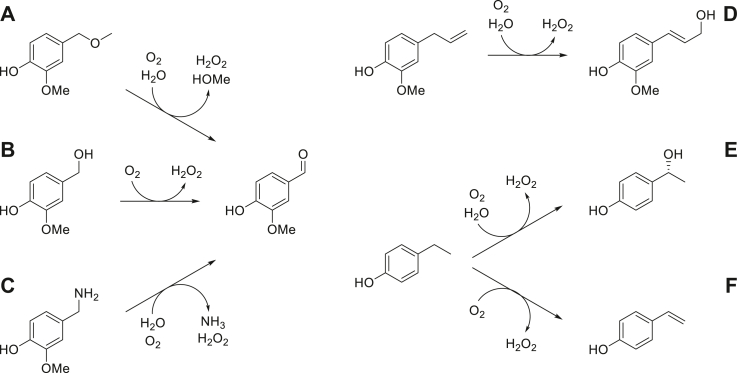
**Reactions catalyzed by*Ps*VAO.***A*, oxidative ether cleavage. *B*, alcohol oxidation. *C*, oxidative deamination. *D*, ɣ-hydroxylation. *E*, α-hydroxylation. *F*, dehydrogenation. Molecular oxygen is always employed as electron acceptor. Reactions (*E* and *F*) are performed in parallel resulting in a product mixture where the ratio of alcohol and alkene products depends on the amino acid composition in the catalytic center. VAO, vanillyl alcohol oxidase.

The elucidation of the enzymatic mechanism revealed that in a first step, the covalently bound flavin adenine dinucleotide (FAD) cofactor is reduced by a hydride transferred from the phenolic substrate (11, 19). As a consequence, a quinone methide intermediate is formed, which is a strong electrophile for the addition of a water molecule. Lastly, FAD is regenerated by molecular oxygen, yielding hydrogen peroxide as byproduct (2).

Despite the high promiscuity of *Ps*VAO, little attention was drawn to other members of this enzyme family which is partially due to difficulties in expression and purification procedures in heterologous hosts, yielding dysfunctional enzymes trapped in an intermediate oxidation state of the covalent flavin cofactor (20). Yet, we decided to continue investigations on this enzyme family since the complexity of lignin degradation in dependency of the type of wood and the respective habitat of each fungus gives valid reason to expect larger functional diversity among other members than a single enzyme can show. We focused especially on ether cleavages as ethers in general, and the β-O-4 bond in particular, are the most abundant linkages in lignin (21). Besides, ether cleavage itself is challenging from a chemical perspective and often requires harsh methods; therefore, we figured a deeper look into nature’s toolbox may reveal potential biocatalysts.

In this work, we systematically evaluated VAO sequences to obtain candidates with altered amino acid compositions in the catalytic center, compared to *Ps*VAO, intending to find novel catalytic features. Based on this sequence analysis, we selected and successfully produced the VAO from *Diplodia corticola* (*Dc*VAO). This ascomycete fungus is called the “bot canker of oak” and represents a plant-pathogen widespread in the eastern US and south-western Europe, responsible for infection of members of red and white oaks including the cork oak (*Quercus suber*) (22, 23). As *Dc*VAO contains a markedly different amino acid composition in the catalytic center compared to *Ps*VAO, we figured it might be an interesting candidate to get insight into the overall enzyme family and their potential role in plant biomass degradation. Therefore, we studied the substrate scope and kinetic parameters of *Dc*VAO and generated two point mutations to investigate the role of Glu466 in the catalytic center. Further, we performed an evolutionary analysis and resurrect the common ancestor of the whole clade of fungal VAOs plus some of the internal nodes. By this, we aimed to disclose the catalytic potential of the complete group.

## Results

### Genome mining and phylogenetic analysis allow identifying enzymes with altered amino acid compositions in the catalytic center

**Figure 2:**
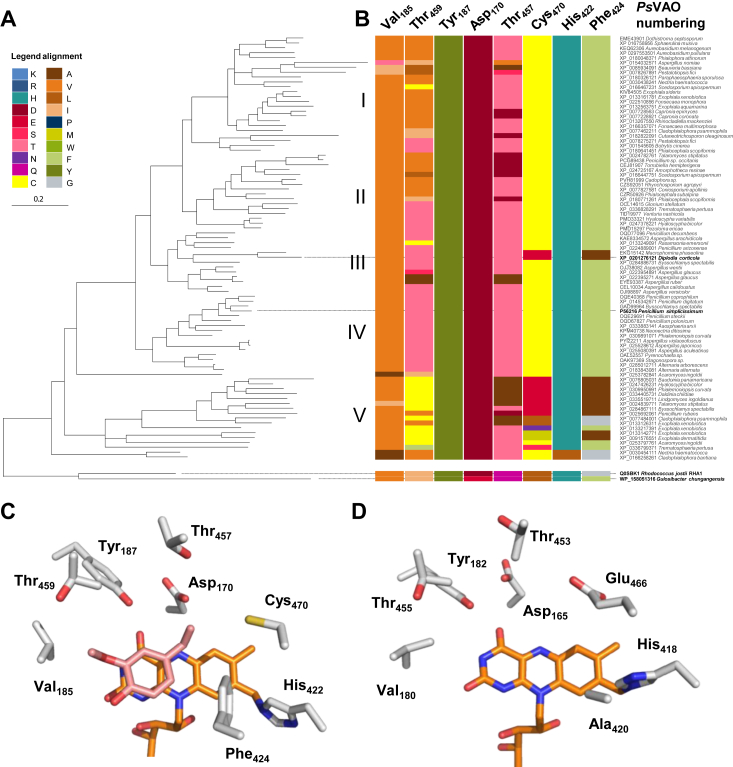
**Natural diversity of residues of the catalytic pocket in vanillyl alcohol oxidases.***A*, phylogenetic tree of fungal VAOs with EUGO from *Rhodococcus jostii* RHA1 and 4EPO from *Gulosibacter chungangensis* as outgroup. Enzymes investigated in this work are shown with names in *bold*: VAOs from *Diplodia corticola* (*Dc*VAO) and from *Penicillium simplicissimum* (*Ps*VAO) respectively. The tree is divided in five clades (I to V). *B*, color-coded amino acids of the catalytic center from *Ps*VAO extracted from the multiple sequence alignment. Numbering corresponds to *Ps*VAO. *C*, key amino acids of the catalytic center of *Ps*VAO are depicted as *sticks*. The figure is based on a crystal structure (PDB: 2VAO). The FAD cofactor is shown in *orange*, and the bound substrate 4-ethylguaiacol is shown in *pink*. *D*, key amino acids of the catalytic center of *Dc*VAO are depicted as *sticks*. The figure is based on a homology model of the enzyme. The FAD cofactor is shown in *orange*. 4EPO, 4-ethyl phenol oxidase; EUGO, eugenol oxidase; FAD, flavin adenine dinucleotide; VAO, vanillyl alcohol oxidase.

The sequence of *Ps*VAO was used to perform homology searches in fungal genomes resulting in about 200 sequences which cluster into five different clades (I to V)(Fig. 2*A*). For a prediction of altered characteristics, we inspected the key residues in the catalytic center of *Ps*VAO in the multiple sequence alignment of the whole data set (Fig. 2*B*). Val185, Thr459, and Tyr187 (*Ps*VAO numbering) restrict the catalytic pocket at the side of the of the substrate entry tunnel, while Tyr187 has been hypothesized to work as the gatekeeper for oxygen access (24). Asp170 is an essential residue for the substrate deprotonation in benzylic position and assists in covalent flavinylation (25, 26). Thr457 and Cys470 are as well in close proximity to the benzylic position of the substrate molecule (Fig. 2*C*) and probably modulate the overall reactivity. In the crystal structure of *Ps*VAO (PDB: 2VAO), *Rj*EUGO (5FXE), and *Gc*4EPO (7BPI), His422 (*Ps*VAO numbering) is responsible for a covalent linkage of the FAD cofactor to the enzyme and restricts, together with Phe424, the catalytic pocket at the opposing side of the substrate entry tunnel (5, 16, 27). Notably, this Phe is replaced by a Gly residue in *Rj*EUGO (27). With the exception of two enzymes, His422 is conserved among all the sequences analyzed. Further, the catalytic triad formed by Tyr108, Tyr503, and Arg504, which is responsible for the binding of the phenol group of the substrate, is also conserved among all VAOs (data not shown) (16, 28). The same is true for Tyr187 and Asp170, which were both described as essential for the reaction mechanism. Differences in the family are rather found for secondary interactions with the substrate. Here, position 459 stands out as fairly variable. Thus, clade III and IV (*Ps*VAO) are distinct from the other clades by the presence of a Thr in position 459 over predominantly Val in clade I and Cys in clade V. In clade II, Thr and Val are common, while this clade also differs from clade III and IV by the occurrence of Glu in position 457 over otherwise Thr. In clade V, Ala is mainly found at this site.

Most important interaction points of substrates with groups *ortho* to the phenolic group are residues in position 424 and 185 (*Ps*VAO numbering). Among the VAO group, the combination of Phe and Val is by far the most common. For position 424, exceptions are found in clade III and V. In clade III, only two VAOs, from *D. corticola* (*Dc*VAO) and *Macrophomina phaseolina* (*Mp*VAO) respectively, contain an Ala in position 424. In contrast, Ala, Gly, or Phe are found for enzymes of clade V with decreasing abundance. The introduction of another amino acid in position 424 often coincides with an amino acid change in position 470. These changes are found most of the time in clade V. In fact, *Dc*VAO and *Mp*VAO are the only two VAOs outside of clade V, which do not contain a Cys in position 470. In general, the combination of Ala424 and Glu470 is found predominantly, but Leu, Met, or in single cases Ser and Asn are also observed in position 470.

The combined occurrence of both amino acid variations raised the question how the catalytic properties of the respective enzymes are affected and which factors caused this unusual variation. Therefore, the habitats of selected fungi were included into the considerations. In this regard, *D. corticola* and *M. phaseolina* are both known plant pathogens with an overlap in habitat on oak trees (23, 29). A similar habitat was observed for *Daldinia childiae*, which is a member of clade V, and also contains the amino acid combination of Glu470 and Ala424 (according to the homologous positions in *Ps*VAO). Thus, it is possible that the Phe/Ala exchange alters the catalytic pocket to accommodate for 2,6-dimethoxylated substrates as they occur in syringyl-rich S-lignin found in hardwood. As we intended to test this hypothesis, *Dc*VAO turned to our focus as *D. corticola* inhabits a narrow habitat of hardwood oaks. Further, the Cys/Glu exchange in position 470 alone is a remarkable change in the catalytic pocket (Fig. 2*D*), and we were interested if this residue influences the substrate scope of the enzymes. Interested in ether cleavages, we expected an altered performance as Glu470 could act as additional proton shuttle. Based on these assumptions, we selected *Dc*VAO for further experimental characterization.

### *Dc*VAO can be produced heterologously in *Escherichia coli* as an FAD-saturated enzyme

The *vao* gene from *D. corticola* was acquired as a DNA fragment *via* gene synthesis and was successfully cloned into the pET16bp vector using the Gibson assembly method. Difficulties in producing fungal VAOs in bacterial hosts were described before and we also experienced issues in that aspect. Therefore, we tested four different *E. coli* expression strains and confirmed the production of the enzyme by formation of vanillin and coniferyl alcohol in a whole-cell assay. *E. coli* NiCo21 (DE3) pKJE7 emerged as best suited host, which encodes for three additional chaperones on the pKJE7 plasmid to promote correct folding of the protein (30, 31). Overexpression was performed at 4.5 l scale in a Braun Biotech bioreactor, and the obtained cells were used to provide a crude extract for subsequent protein purification. After His-tag affinity chromatography, a yellow enzyme was obtained with a yield of 9.1 mg per l broth. As reference, we also produced *Ps*VAO as described earlier (32).

Spectral analysis of *Dc*VAO showed three peaks at 273, 360, 441 nm, whereof the peak at 360 nm is characteristic for a covalent-bound flavin cofactor (Fig. S1) (33). We obtained a ratio 280 to 441 nm of ∼9.4, which indicates full cofactor saturation. This was validated by comparison of the total protein content determined by Bradford analysis and the concentration for the FAD cofactor using the absorption at 441 nm (ε = 14,200 M^−1^ cm^−1^). Size-exclusion chromatography revealed the protein to occur in a dimeric and an octameric complex (Fig. S2). The same oligomerization state was described for *Ps*VAO which was attributed to the presence of a so-called octamerization loop which is present for all VAOs but absent for exclusively dimeric EUGOs and 4EPOs (34). Our results strengthen this hypothesis. *Dc*VAO was found to be stable up to a temperature of 50 °C after an incubation of 2 h at the respective temperature (Fig. S3). Stability across a broad pH range from 6.5 to 9.5 (Fig. S4) was observed. No activity could be determined at pH values of 4.5 and below, while at pH of 10.5, a remaining activity of 13% was found.

### *Dc*VAO accepts 2,6-dimethoxylated substrates and shows high activity for ether cleavages

**Figure 3:**
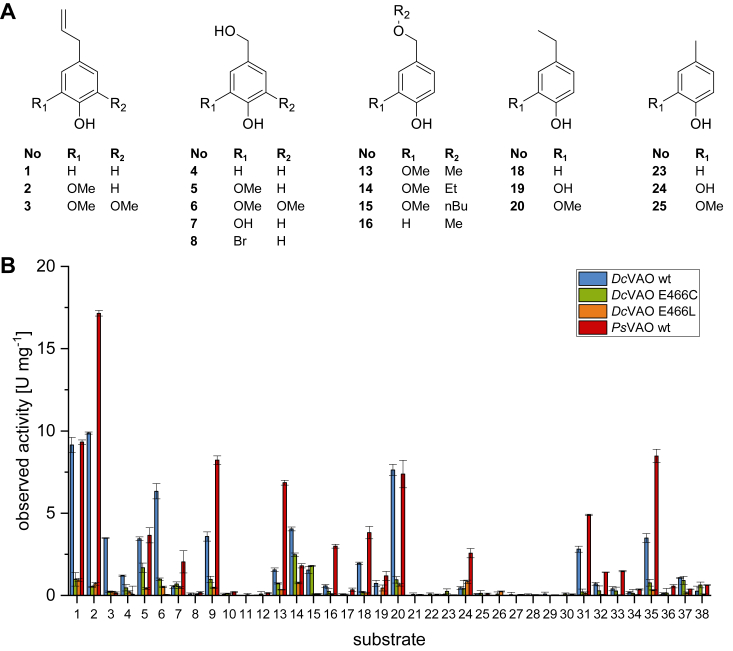
**Substrate scope of *Dc*VAO WT and variants in comparison with *Ps*VAO WT.***A*, overview of the chemical structures of selected substrates. For the complete list, see Table S1. *B*, substrate selectivity of *Dc*VAO WT and variants compared to *Ps*VAO WT. The reactions were performed with 50 nM of enzyme in potassium phosphate buffer at pH 7.5 and 25 °C. Product formation was determined by the hydrogen peroxide production in a colorimetric assay. VAO, vanillyl alcohol oxidase.

Enzyme activity for *Dc*VAO and *Ps*VAO were measured in an endpoint kinetic assay, determining the hydrogen peroxide concentration in the solution (xylenol orange assay) (10). Product formation was validated by GC-MS. In total, 38 substrates were tested and both enzymes were found to be active on about two thirds of them (*Dc*VAO: 23, *Ps*VAO: 24) (Fig. 3 and Table S1). As described in literature, *Ps*VAO was found to perform hydroxylations in α- and ɣ-position, oxidation of benzyl alcohols, dehydrogenation, as well as oxidative ether cleavages. The same reaction pattern was also found for *Dc*VAO (Table S2). Similar to *Ps*VAO, we detected a preference for hydroxylations on substrates (Figs. S5–S7). *Dc*VAO performed the hydroxylation of 1,2-bicyclic substrates and ethyl phenol derivatives, while for 4-cyclopentyl phenol (**35**), only dehydrogenation products were observed. Both enzymes performed rather poorly on deamination reactions, although *Ps*VAO has been described to be capable of performing this type of reaction (14). Notable differences between the two enzymes were observed with regard to the substitution pattern *ortho* to the phenol moiety, as *Dc*VAO was found to accept 2,6-dimethoxylated substrates. Activities of 3.5 ± 0.02 U mg^−1^ and 6.3 ± 0.5 U mg^−1^ on 4-allyl-2,6-dimethoxy-phenol (**3**) and 4-(hydroxymethyl)-2,6-dimethoxyphenol (**6**) were observed, respectively, while for *Ps*VAO, only residual activities <0.15 U mg^−1^ were detected. Further, *Dc*VAO achieved high conversions on vanillyl ether derivatives with activities up to 4.0 ± 0.1 U mg^−1^ for ethyl vanillyl ether (**14**). We further investigated this behavior by kinetic studies for both enzymes. The K_M_ value for eugenol is similar for both enzymes, and a cooperative effect according to Hill was observed for both as well (Table 1 and Fig. S8). The cooperative effect is less pronounced for *Dc*VAO (n = 2.6 ± 0.2) as for *Ps*VAO (n = 4.8 ± 0.6), which cannot be fully explained yet as both enzymes were shown to have the same multimerization state. It is likely that the substrate inhibition observed for *Ps*VAO at high concentrations (K_i_ = 12.3 ± 4.5 mM) contributes to this effect. No inhibition was observed for *Dc*VAO. Further, *Dc*VAO has an about two-fold increased K_M_ values for vanillyl alcohol and vanillyl ether derivatives and was found to be more active on vanillyl ethyl (**14**) and vanillyl butyl ether (**15**) (Figs. S9–S11). For the later, no activity was observed for *Ps*VAO while *Dc*VAO had a turnover frequency of 2.1 ± 0.1 s^−1^ (Fig. S12). On the other hand, *Ps*VAO was found to be more active on vanillyl methyl ether (**13**).

**Table 1:** Kinetic data from Michaelis–Menten experiments for *Dc*VAO and *Ps*VAO

Substrate	No	Enzyme	Equation	K_M_ [μM]	*k*_cat_ [s^−1^]	n	K_i_ [mM]
Eugenol	**2**	*Dc*VAO	(2)	60 ± 3	10.3 ± 0.1	2.6 ± 0.2	-
		*Ps*VAO	(4)	67 ± 2	18.7 ± 0.5	4.8 ± 0.6	12.3 ± 4.5
Vanillyl alcohol	**5**	*Dc*VAO	(1)	297 ± 21	4.0 ± 0.1	-	-
		*Ps*VAO	(1)	144 ± 10	6.7 ± 0.1	-	-
Vanillyl methyl ether	**13**	*Dc*VAO	(1)	263 ± 12	1.9 ± 0.01	-	-
		*Ps*VAO	(1)	155 ± 5	7.9 ± 0.1	-	-
Vanillyl ethyl ether	**14**	*Dc*VAO	(1)	198 ± 19	4.7 ± 0.2	-	-
		*Ps*VAO	(1)	86 ± 3	2.6 ± 0.02	-	-
Vanillyl butyl ether	**15**	*Dc*VAO	(1)	258 ± 27	2.1 ± 0.1	-	-
		*Ps*VAO	-	n.d.	n.d.	n.d.	n.d.

The reactions were performed at 25 °C in potassium phosphate buffer at pH 7.5. All ether substrates and vanillyl alcohol follow a classical Michaelis–Menten kinetic mechanism, while a cooperative effect was observed for substrate eugenol. Further, *Ps*VAO displays substrate inhibition. Subsequently, the kinetic data for eugenol with *Dc*VAO was fitted with the Hill equation (52) and the kinetic data for *Ps*VAO was fitted with the Licata equation (53). For further details, see Figs. S8–S12 in the section Michaelis-Menten kinetics for DcVAO and PsVAO in the Supporting information. Numbers in bold indicate substrate numbers.

The Michaelis–Menten kinetics for *Dc*VAO and *Ps*VAO showed that *Dc*VAO has overall higher K_M_ values for all substrates except for eugenol. Regarding the *k*_cat_, *Ps*VAO has a higher turnover frequency on eugenol (**2**), vanillyl alcohol (**5**), and vanillyl methyl ether (**13**), while *Dc*VAO is twice as active on vanillyl ethyl ether (**14**) and the only active enzyme on vanillyl butyl ether (**15**). This highlights a preference of *Dc*VAO for longer chains compared to *Ps*VAO.

As the highest activity of *Dc*VAO was observed for vanillyl ethyl ether, also the highest total turnover number (>13,500 TTN) was reached for this substrate, while for vanillyl methyl and butyl ether, a lower initial rate and a lower TTN was observed (>9200 and >7300 TTN, respectively, Fig. S13).

### Structural analysis reveals key residues influencing the substrate selectivity

*Dc*VAO was found to accept 2,6-dimethoxylated substrates and to be highly active on vanillyl ethyl derivatives. In order to investigate the molecular origins for these characteristics, we built a homology model for *Dc*VAO based on the *Ps*VAO structure (PDB 2VAO). In addition, a *de novo* model with AlphaFold2 was generated and compared to the previously obtained homology model and the crystal structure of *Ps*VAO. The three structures aligned well, so that we decided to continue with the homology model for further calculations. Subsequently, a structure-guided analysis of the catalytic center by docking several substrates to the catalytic cavity of *Dc*VAO was performed and substrate interactions with the amino acids of the active side were evaluated. Although the majority of amino acids is the same for both enzymes (Fig. 2, *C* and *D*), notable differences were observed in the positioning of the substrate molecule: while in *Ps*VAO, the methoxy group of 4-ethylguaiacol is directed towards Val185, all docked substrates are turned in *Dc*VAO (Fig. S14) and for vanillyl ethyl ether, the single methoxy group is rather pointed in the direction of Ala420 (Fig. S15). As already pointed out in the phylogenetic analysis, most VAO sequences contain a Phe in position 424, while *Dc*VAO contains an Ala instead (A420). This amino acid change widens the catalytic pocket and generates space for 2,6-dimethoxylated substrates (Figs. S16 and S17), which is in agreement with the higher K_M_ values observed for *Dc*VAO on 2-methoxylated substrates. It can be speculated, if this amino acid variation is related to a habitat containing high amounts of syringol (4-hydroxy-3,5-dimethoxycinnamic alcohol), as it is the case for *D. corticola* or if other structural reasons are the determining factor. Nevertheless, the additional space in the catalytic pocket is likely the reason for single methoxylated substrates to adopt a different binding pose compared to *Ps*VAO.

**Figure 4:**
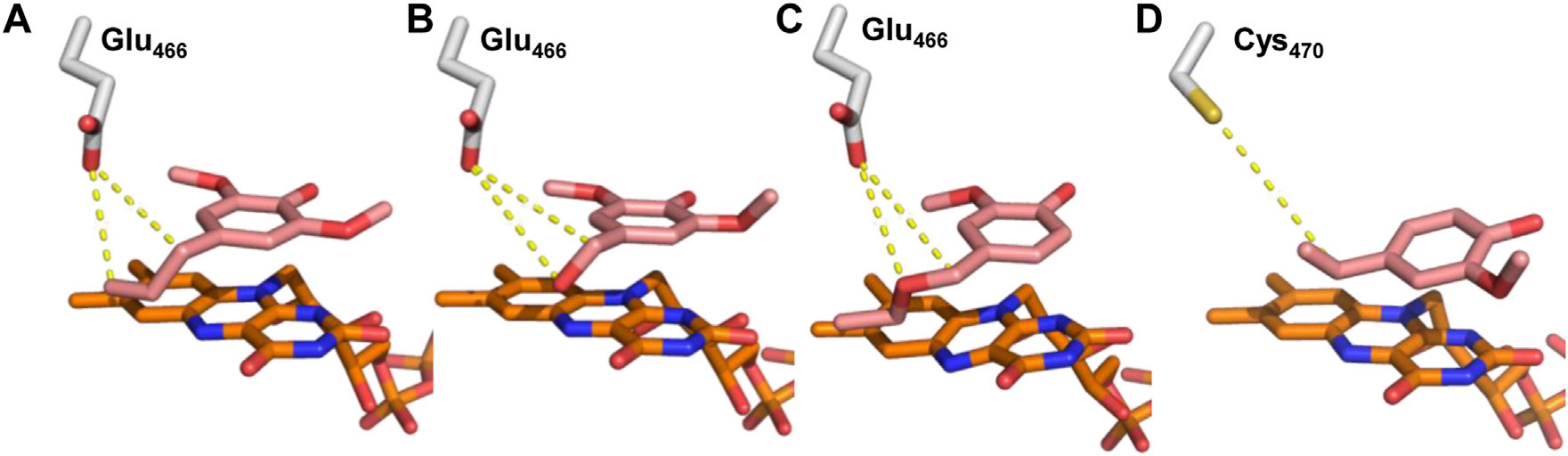
**Distances of Glu466 to indicatedatomsof respective substrate molecules in the homology model of *Dc*VAO after docking and subsequent energy minimization by molecular dynamics simulations.***A*, for 4-allyl-2,6-dimethoxy-phenol (**3**), the distance from the oxygen atom to the C_ɣ_ atom is half an angstrom shorter than to the C_α_ atom (O_Glu_-C_α_: 4.2 Å, O_Glu_-C_ɣ_: 3.7 Å). The O_Glu_-C_ɣ_ distance is the closest distance measured for any of the substrates. *B*, for 4-(hydroxymethyl)-2,6-dimethoxyphenol (**6**), the distances towards the C_α_ atom and the benzylic oxygen are the same (O_Glu_-O_β_: 4.8 Å, O_Glu_-C_α_: 4.8 Å). *C*, for vanillyl ethyl ether (**14**), a closer distance towards the ether oxygen is observed than to the C_α_ atom (O_Glu_-C_α_: 4.7 Å, O_Glu_-O_β_: 4.1 Å). *D*, distance in the *Ps*VAO crystal structure (2VAO) between the sulfur atom of Cys470 to the C_α_ atom of the bound substrate molecule 4-ethylguiacol (S_Cys_-C_α_: 5.5 Å). In general, the distance in *Ps*VAO is much larger and beyond hydrogen bond distance compared to *Dc*VAO. VAO, vanillyl alcohol oxidase.

In position 466 (470 in *Ps*VAO), *Dc*VAO features a Glu over the more common Cys among other members of the family. This residue is located in close proximity to the *para* residue of the substrate. Docking of 4-allyl-2,6-dimethoxy-phenol (**3**) revealed that Glu466 is also likely to favor ɣ-hydroxylations as the O_Glu_-C_ɣ_ distance was determined to be 3.7 Å (Fig. 4*A*). For 4-(hydroxymethyl)-2,6-dimethoxyphenol (**6**), a distance of 4.8 Å was found for the O_Glu_-O_β_ and O_Glu_-C_α_ interactions (Fig. 4*B*). Similar values were measured for the O_Glu_-C_α_ distance (4.7 Å) in the *Dc*VAO homology model docked with vanillyl ethyl ether (**14**), while the O_Glu_-O_β_ distance was 0.6 Å shorter (4.1 Å)(Fig. 4*C*). In contrast, in the crystal structure of *Ps*VAO (PDB 2VAO), a S-C_α_ distance of 5.5 Å was measured (Fig. 4*D*). The positioning of Glu466 leaves the residue in a prime position to possibly act as a proton shuttle for hydroxylation reactions and may also facilitate ether cleavages. To investigate this hypothesis, we replaced Glu466 by a Cys and a Leu in *Dc*VAO. Cys is the most common amino acid for related VAOs and also present in *Ps*VAO, while Leu occurs naturally in EUGOs and 4EPOs for which little to no activity for ether cleavages was reported (0.004 s^−1^ for *Rj*EUGO) (7).

### Variation of the residue in position 466 is probably a result from the repositioning of the substrate molecule

*Dc*VAO variants were generated by means of the QuikChange method, and proteins were produced according to the same protocol as for the WT enzyme. In comparison, the variants were produced in lower yields (*Dc*VAO E466C: ∼3 mg l^−1^, *Dc*VAO E466L: ∼1 mg l^−1^). The substrate scope of the variants was investigated by using the same endpoint kinetic measurements as described before. In total, 33 substrates were tested as we excluded five from the initial set since no conversion was observed neither for *Dc*VAO or *Ps*VAO. For *Dc*VAO E466C and E466L, a significantly altered substrate profile was found: *Dc*VAO E466C was found to retain 50 to 100% activity for the cleavage of vanillyl ethers where the percentage increased with the chain length (Fig. 3*B*). Unchanged conversion was observed for 5-hydroxyindane (**37**), 5,6,7,8-tetrahydro-2-naphtol (**38**), and 3,4-dihydroxybenzyl alcohol (**7**). For other benzyl alcohol derivatives, like vanillyl alcohol (**5**), the residual activity was 49%. A stronger reduction in activity was observed for hydroxylation reactions. 4-Ethylguaiacol (**20**) represents the only converted ethyl phenol derivative with a residual activity of 12%. Even lower activities were observed for the hydroxylation of allyl phenol derivatives resulting for this group in the overall strongest decrease.

For *Dc*VAO E466L, an overall reduced activity was found as well. For *p*-allyl substrates, a more than ten-fold reduction was observed, while for *p*-benzyl alcohol derivatives, activities of 17% and lower were found compared to the WT enzyme. A residual activity of about 20% was found for vanillyl methyl (**13**) and ethyl ether (**14**), while no conversion could be detected for vanillyl butyl ether (**15**). The highest activity on ether substrates was observed for the ethyl derivative with 0.76 ± 0.05 U mg^−1^. Notably, a comparably lower reduction was found for catechol-like substrates. For 3,4-dihydroxybenzyl alcohol (**7**) and ethyl catechol (**19**), the residual activity of 90% and 61%, respectively, is rather high compared to other substrates. In case of methyl catechol (**24**), an activity of 0.83 ± 0.10 U mg^−1^ was observed, which represents an increase of 182% compared to the WT. As *Ps*VAO is active on catechol-like substrates as well, it can be hypothesized that a hydrophobic interaction next to His422 (*Ps*VAO numbering) might be beneficial for acceptance of these substrates. In case of *Dc*VAO E466L, the respective Leu would perform this task, while Phe424 interacts with the substrate in *Ps*VAO.

The tendency in decreasing activity from the WT over the E466C to the E466L variant highlights the importance of a polar interaction from residue 466 with the substrate in *Dc*VAO. The strongest impact was found for ɣ-hydroxylations, which is in good agreement with the close O_Glu_-C_ɣ_ distance. These results contrast with observations for *Ps*VAO, as no impact on the activity on eugenol (**2**) or vanillyl alcohol (**5**) were observed in earlier studies with the C470L variant (10). But as *Ps*VAO acts on *p*-allyl phenols with up to 17.2 ± 0.2 U mg^−1^ for eugenol (**2**) and contains a Cys in the respective position, clearly other factors determine the substrate acceptance in *Dc*VAO. The changed positioning of the substrate observed in the *in silico* analysis is the most likely explanation for this (Fig. S14). This raised the question about when and how the change in substrate orientation occurred. We therefore decided to resurrect three ancestral enzymes to attempt responding this.

### Resurrection of ancestral VAOs helps in rationalizing substrate selectivity

**Figure 5:**
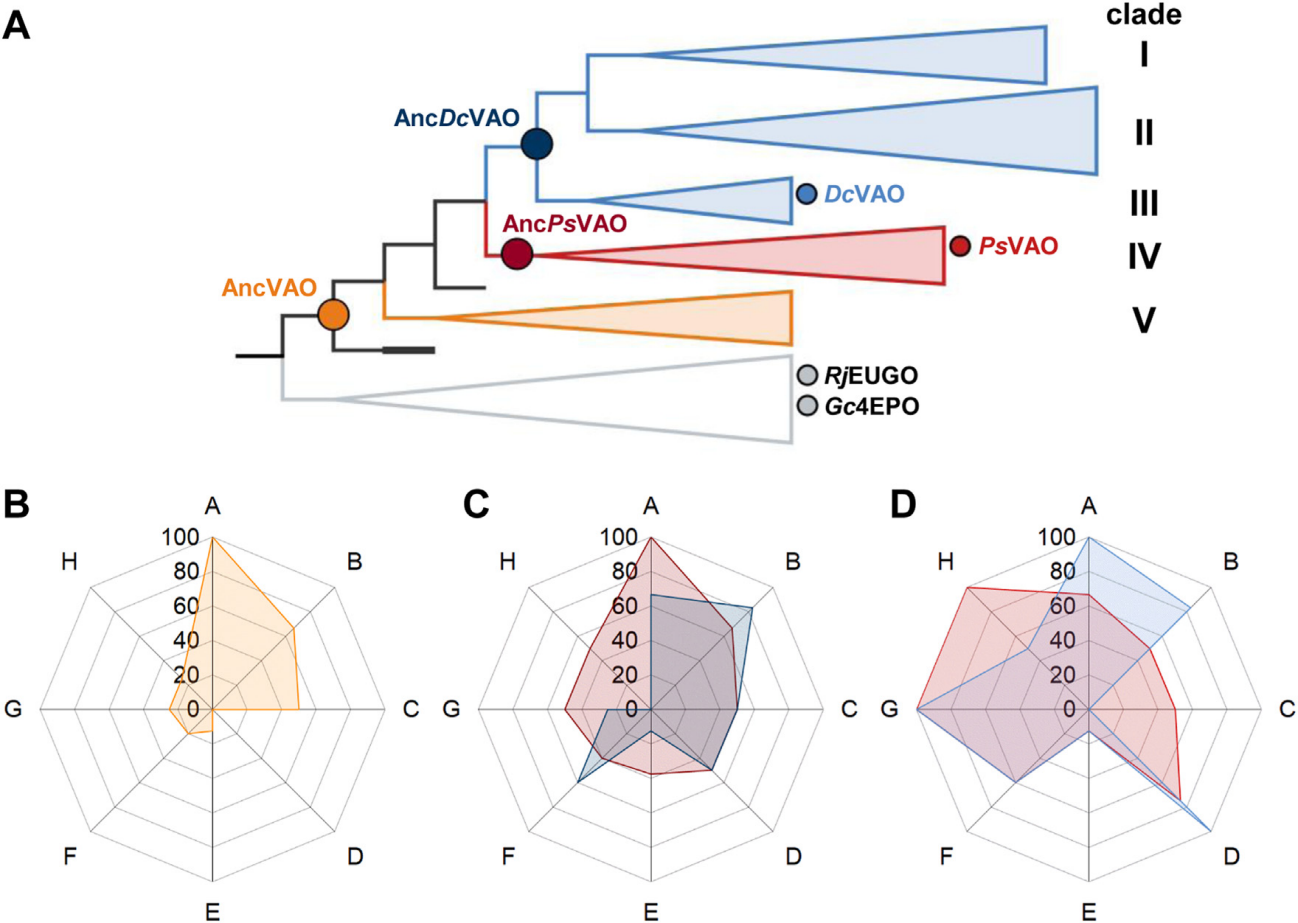
**Development of the substrate selectivity from resurrected ancestral enzymes to modern enzymes.***A*, compressed phylogeny employed for ASR. The VAO group is shown with *black branches*. The clade IV including *Ps*VAO in shown in *red*, while clades I, II, and III (this including *Dc*VAO) in *light blue* and uncharacterized clade V is shown in *orange*. Bacterial EUGO & 4EPO homologs were used as outgroup (shown in *gray*). The resurrected ancestors are marked with *larger dark colored circles* and the extant sequences with *smaller and lighter ones*. All circles are colored according to their clades. *B*, substrate profile of AncVAO. The percentage of converted compounds of the respective categories (*A*–*H*) (see below) is shown. *C*, substrate profile overlap of Anc*Ps*VAO (*red*) and Anc*Dc*VAO (*blue*). The percentage of converted compounds of the respective categories (*A*–*H*) is shown. *D*, substrate profile overlap of *Ps*VAO (*red*) and *Dc*VAO (*blue*). The percentage of converted compounds of the respective categories (*A*–*H*) is shown. Substrates were considered transformed when the observed activity on a substrate was higher than the 7.5% of the average activity of all converted substrates from the respective enzyme. In total, 37 substrates were tested which were grouped in eight categories by chemical characteristics. Substrate categories share a common structural motif and differ in other regarding the chain length or *o*-substitution. Categories: *p*-allyl phenol (A), *p*-benzyl alcohol (B), *p*-benzyl amine (C), *p*-benzyl ether (D), *p*-methyl phenol (E), *p*-ethyl phenol (F), *p*-alkyl phenol (G, chain length > 2), bicyclic phenol (H). For further details, see Table S3. 4EPO, 4-ethyl phenol oxidase; EUGO, eugenol oxidase; VAO, vanillyl alcohol oxidase.

To determine the potential substrate scope of the VAO family as well us to understand the sequence determinants of it, ancestral reconstruction was conducted. The common ancestors of the clades including *Ps*VAO (Anc*Ps*VAO, $\bar{PP}:0.94$) and the one including *Dc*VAO (Anc*Dc*VAO, $\bar{PP}:0.94$) were reconstructed along with the ancestor of the whole group (AncVAO, $\bar{PP}:0.87$)(Fig. 5*A*). Genes of ancestral enzymes were ordered as synthesized gene fragments, cloned, and expressed in the same way as for the *Dc*VAO gene. Enzyme yields were generally lower than *Dc*VAO. AncVAO and Anc*Ps*VAO were produced with 2.3 and 2.2 mg per l broth, respectively, while 0.3 mg per l broth of Anc*Dc*VAO were obtained.

In total, 37 substrates were tested for conversion by the ancestral enzymes (Table S3). To better trace changes in the selectivity of each enzyme during evolution, the substrates were grouped into eight categories of chemically similar compounds. The percentage of converted substrates for each category gives the substrate profile of the enzyme (Fig. 5*B*). Ultimately, homology models were calculated for the ancestral proteins to correlate the substrate profile with changes in the amino acid composition.

Over the course of evolution, it seems plausible to speculate that the substrate profile of VAOs first diversified and later specialized as it is the case of modern enzymes. AncVAO shows a narrow selectivity (12 compounds) towards *p*-allyl phenols, benzyl alcohols, and amines, while ether and alkyl substrates were rarely accepted (Fig. 5*B*). The highest activities of ∼1 U mg^−1^ are observed for chavicol (**1**), eugenol (**2**), vanillyl alcohol (**5**), and 4-(1-hydroxyethyl)phenol (**9**). As this enzyme is the closest relative to EUGOs and 4EPOs, it is not surprising that the observed substrate profile is similar to that of *Rj*EUGO, which also performs poorly in ether cleavage reactions and hydroxylations of cresol derivatives (10). This is also reflected in the structural model (Fig. S18*A*): Like *Rj*EUGO, AncVAO harbors a Gly in position 424 and a Val in position 459 (*Ps*VAO numbering). Docking experiments revealed that these residues cause the model substrate eugenol to adopt an alternative binding position which again is comparable to the binding mode observed in the crystal structure of *Rj*EUGO (Fig. S18*B*). Thus, the positioning of the substrate might be the reason for the EUGO-like substrate scope of AncVAO. The fact that AncVAO contains a Cys in position 470 (PsVAO numbering) while in the structure of *Rj*EUGO a Leu is found seems not to influence the substrate scope.

Anc*Ps*VAO and Anc*Dc*VAO have both a broader substrate scope than AncVAO (19 and 16 compounds, respectively) but do not accept many substrates in the respective categories where *p*-allyl phenols and benzyl alcohols are the exception. Both enzymes contain a Phe in position 424 (*Ps*VAO numbering), and docked eugenol adopts a similar binding mode as observed for *Ps*VAO (Figs. S19 and S20*A*). Further, Anc*Ps*VAO and Anc*Dc*VAO feature a Cys in position 470. With regard to the reaction profile, first tendencies are visible which carry over to the clades later on: Anc*Ps*VAO converts bicyclic compounds and such with longer alkyl chains. Also, the ether cleavage reaction is introduced in the reaction profile, which makes Anc*Ps*VAO the most promiscuous ancestral enzyme. *Ps*VAO substrate profile contains 22 converted substrates where a clear selectivity towards longer chains in *para*-positions can be observed (Fig. 5, *B* and *C*). While 40 to 50% of *p*-alkyl substrates (chain length >1) were accepted by Anc*Ps*VAO, the acceptance increases to 60% for *p*-ethyl to 100% for *p*-alkyl substrates with a chain length >2 in *Ps*VAO. Further, all cyclic substrates were accepted, which contain a steric demanding group in *para*-position as well. Anc*Ps*VAO is the only ancestor converting 4-hydroxy benzyl methyl ether, indicating that this selectivity was adopted for clade IV during evolution, thus strengthening the assumption that this compound might be the natural substrate for *Ps*VAO (1). For *p*-allyl phenols, benzyl alcohols, and amines, the acceptance of *Ps*VAO remained similar to Anc*Ps*VAO. Structure-wise, the catalytic center of Anc*Ps*VAO and *Ps*VAO contain the same amino acid composition (Fig. S19), which is in agreement with the similar substrate profile. As a consequence, the higher activity observed for *Ps*VAO might be due to different structural reasons.

For *Dc*VAO, the same tendency for the chain length of *p*-alkyl substrates was observed as for *Ps*VAO. But apart from this, the enzyme took a different path. *Dc*VAO is rather versatile in ɣ-hydroxylations and the oxidation of benzyl-alcohol derivatives as the enzyme tolerates many residues in the *ortho*-positions of the substrate. This is partially reflected in clade III as Anc*Dc*VAO was found to accept a variety of benzyl alcohols with activities ranging between <0.1 to 0.31 U mg^−1^. This includes 3-bromo-4-hydroxy benzyl alcohol (**8**, 0.16 ± 0.04 U mg^−1^), which was not converted by any other ancestral enzyme. Despite reasonable acceptance for *p*-allyl substrates, observed activities for Anc*Dc*VAO were below 0.1 U mg^−1^ for eugenol (**2**) and 4-allyl-2,6-dimethoxy-phenol (**6**). As Anc*Dc*VAO possesses a Cys in position 466 (*Dc*VAO numbering), the enzyme is more comparable to the *Dc*VAO E466C variant (Fig. S20). Thus, the low activity on allyl phenols is in agreement with the mutagenesis studies. Further, Anc*Dc*VAO is the most active ancestral enzyme on vanillyl ethyl ether (**14**) being the only one converting vanillyl butyl ether (**15**)(0.23 ± 0.16 and 0.29 ± 0.11 U mg^−1^, respectively). This selectivity is in line with the substrate scope of wt *Dc*VAO and E466C, which both accepted 100% of all benzyl ether compounds. A notable difference between Anc*Dc*VAO and *Dc*VAO is the ability of the ancestral enzyme to perform deamination reactions. As this activity could be restored for *Dc*VAO E466C on 4-(1-aminoethyl)phenol (**10**)(0.1 ± 0.03 U mg^−1^), it is tempting to attribute the loss of this reaction type to the introduction of Glu466 which would strongly interact with the amine group. Moreover, Glu466 seems to be mainly responsible for hydroxylation reactions in α- and ɣ-position as Anc*Dc*VAO and *Dc*VAO E466C were found to possess only residual activities on those substrates. But Glu466 is not solely responsible for the oxidation of alcohol or the cleavage of ether. For the later, the reducing effect in activity can even be offset by longer chain length in *para*-position as observed for butyl vanillyl ether (**15**). This is supported by *in silico* experiments as the distance between the reactive oxygen of Glu466 is closer to the ɣ-carbon atom than to the α-carbon atom of the substrate molecule. Another factor is the orientation of the substrate molecule in the catalytic pocket. Due to the introduction of Ala420, additional space is generated causing the substrates to rotate compared to *Ps*VAO (Fig. S14). Thus, it is likely that the Cys/Glu exchange is necessary to retain hydroxylating activity after adoption towards 2,6-methoxylated substrates by the introduction of Ala420. This hypothesis is strengthened by the fact that both mutations occur only in combination. Nevertheless, the fact that Anc*Dc*VAO harbors a Phe in the respective position highlights that additional factors in protein folding may play a role as well.

## Discussion

In this study, we describe the second member of the fungal VAO family, the enzyme from *D. corticola*, referred to as *Dc*VAO. The enzyme was selected after phylogenetic analysis of the VAOs. Special attention was drawn to its amino acid composition in the catalytic center. *Dc*VAO differs from the majority of VAO group members in the two positions Ala420 and Glu466 (*Dc*VAO numbering). Ala420 widens the space in the catalytic pocket for substrates with moieties positioned *ortho* to the phenolic group. This became obvious comparing the substrate selectivity of *Dc*VAO with the already described VAO from *P. simplicissimum* (*Ps*VAO) containing a Phe in the respective position, as do most VAOs. *Dc*VAO reached up to 6.3 U mg^−1^ on 2,6-substituted substrates like 4-(hydroxymethyl)-2,6-dimethoxyphenol (**6**), while *Ps*VAO was barely active. Since the Ala exchange occurs naturally combined with the exchange of Cys in position 466, we further investigated this change in expectation of an improving effect on ether cleavages. Mutagenesis studies indicated that the introduced Glu466 in *Dc*VAO is important for hydroxylation in α- and ɣ-position, while ether cleavage and oxidation of benzylic alcohols was reduced but still possible in the E466C variant. The fact that *Ps*VAO is active on these compounds, although containing a Cys in the same position, highlighted that other factors might be involved as well. *In silico* experiments revealed a changed orientation of the substrate molecule in the catalytic pocket of *Dc*VAO compared to *Ps*VAO, which is required to accommodate for 2,6-substituted compounds. As a consequence, Glu466 restores the original substrate scope of the enzyme. This observation was further supported by the analysis of the resurrected ancestors, on which Anc*Dc*VAO has a similar profile to that of *Dc*VAO E466C variant. While Anc*Dc*VAO was found to have low activities in ɣ-hydroxylation reactions, the enzyme was able to perform deamination reactions. This reaction type was absent for *Dc*VAO but could be restored in the *Dc*VAO E466C variant.

It is tempting to attribute the wider catalytic pocket and the acceptance of 2,6-dimethoxylated substrates to the habitat of *D. corticola* as the fungus is a known pathogen to oaks. These trees contain hardwood, which is rich in 2,6-substituted syringyl lignin. Among the phylogenetic tree, some other examples are found for which the fungal habitat coincides with the amino acid composition in the catalytic center: *M.* *phaseolina* (clade III) and *D. childiae* (clade V) are both plant-pathogen infecting oak trees and contain the amino acid combination of Glu470 and A424, like *Dc*VAO (29, 35). Further, plant-pathogenic fungi like *Sphaerulina musiva* or *Dothistroma septosporum* with a habitat on softwood trees like conifers, pine, or poplar are found in clade I and harbor the amino acid combination of Cys470 and Phe424 which are the same as found in *Ps*VAO (36, 37). However, expression of these enzymes and subsequent characterization *in vitro* is required to validate this hypothesis drawn from the sequence data.

To summarize the knowledge obtained about the evolution of the VAO family, we can state that AncVAO shows a substrate profile more similar to that of the bacterial EUGO from *R. jostii* RHA1 and 4EPO from *G. chungangensis* as it could be expected from the evolutionary history. From there on, Anc*Ps*VAO evolved to accommodate substrates with large groups in *para*-position of the phenolic moiety like bicyclic compounds and such with longer alkyl chains. In contrast, Anc*Dc*VAO was found to have a high acceptance towards *p*-allyl, ether, and benzyl alcohol substrates, although the activity for ɣ-hydroxylations is rather poor. These features are in line with the high activity observed for *Dc*VAO on these compounds where ɣ-hydroxylation was restored by the introduction of Glu466. Thus, we observe a varying degree of specialization of VAO enzymes over time, which highlights the catalytic versatility of this protein family.

## Experimental procedures

### Phylogenetic analysis and ancestral sequence reconstruction

Sequence data set was constructed by homology searches in BLASTp using as query *Ps*VAO (Genbank accession P56216). From the raw data set, redundancies and none-fungal sequences were removed. As outgroups, the sequences from EUGO from *R. jostii* RHA1 (*Rj*EUGO) and 4EPO from *G. chungangensis* (*Gc*4EPO) were included. A multiple sequence alignment was constructed by means of the ClustalW algorithm in the Mega 11 software (https://www.megasoftware.net/) (38). A phylogenetic tree was calculated from the alignment using neighbor joining algorithm in Mega 11 and EMBL online services (39, 40).

Ancestral sequence reconstruction was performed in PAMLX v.4.9 as marginal reconstruction using CODEML (41). For this, a maximum likelihood phylogeny was inferred in RaxML v8.2.10, with 500 rapid bootstrapping employing an multiple sequence alignment constructed in MAFFT V7 (292 seqs, 652 sites) (42, 43). Sequences were analyzed using an empirical substitution matrix, an empirical equilibrium amino acid frequencies, four gamma categories, and LG substitution matrix. The posterior probability distribution of the ancestral states at each site was analyzed at nodes corresponding to AncVAO, Anc*Ps*VAO, and Anc*Dc*VAO. The length of the ancestors (AncVAO and Anc*Ps*VAO, 571 amino acids and Anc*Dc*VAO, 570 amino acids) was treated by parsimony analyzing the presence/absence of gaps in the targeted nodes on the basis of the length of the derived sequences in each clade (44). The overall posterior probabilities $\bar{\left(pp\right)}$ for each targeted ancestor were determined as the mean value of the PPs per site. Sites were considered ambiguously reconstructed when the alternative states displayed PP >0.2.

### Homology modeling and structural analysis

For structural analysis of *Dc*VAO, a homology model calculated by YASARA and an AlphaFold2 structure were generated using the sequence from the NCBI database (Accession number XP_020127612) (45, 46, 47). As template for homology modeling, the crystal structure of *Ps*VAO (PDB 2VAO) was used as both enzymes share 62.5% sequence identity. The structures were inspected using PyMol (48). Autodocking was performed in YASARA using the VINA autodock software (https://vina.scripps.edu/) (49). The structures were refined by performing molecular dynamics simulation for 5 ns, using the YASARA2 force field, and creating a water-filled periodic simulation box 5 Å around the structures.

### Gene synthesis and cloning

All sequences were codon optimized for *E. coli* and synthesized as a DNA fragment by Twist Bioscience. The genes were cloned blunt end into pJET2.1 vectors using the New England Biolabs kit. After sequencing, genes were cloned into the pET16 bp vector by means of the Gibson assembly (50). Primers are listed in Table S4. The success of the PCR was controlled by agarose gel electrophoresis. The Gibson assembly mix was digested with DpnI for 1.5 h at 37 °C before being transformed in competent *E. coli* DH5a cells, which were plated on LB agar containing 100 μg ml^−1^ ampicillin. Clones were picked the next day and grown in a 5 ml LB medium (10 g l^−1^ tryptone, 10 g l^−1^ NaCl, 5 g l^−1^ yeast extract, 100 μg ml^−1^ ampicillin) overnight. Plasmids were isolated by the use of Macherey-Nagel NucleoSpin Plasmid Mini Kit, and the success of cloning was validated by sequencing.

### Generation of protein variants

For the generation of *Dc*VAO mutants, QuikChange mutagenesis was applied using the PrimeSTAR Max DNA polymerase from Takara Bio with 25 μl of total reaction volume, 100 ng of pET16 bp_DcVAO plasmid as template, and 10 pmol of the respective primer. Primers were designed for the respective mutation targets as fully and partially overlapping pairs (Table S5). In total, 30 PCR cycles were applied using an annealing temperature recommended for the primer. The success of the PCR was controlled by agarose gel electrophoresis. The PCR product was digested with DpnI for 1.5 h at 37 °C before being transformed in competent *E. coli* DH5a cells, which were plated on LB agar containing 100 μg ml^−1^ ampicillin. Clones were picked the next day and grown in a 5 ml LB medium (10 g l^−1^ tryptone, 10 g l^−1^ NaCl, 5 g l^−1^ yeast extract, 100 μg ml^−1^ ampicillin) overnight. Plasmids were isolated by the use of Macherey-Nagel NucleoSpin Plasmid Mini Kit, and mutations were validated by sequencing.

### Screening of the expression host

In an initial experiment, the plasmid encoding for *Dc*VAO was transformed into *E. coli* Rosetta (DE3), *E. coli* SHuffle (DE3), *E. coli* NiCo21 (DE3) pKJE7, and *E. coli* BL21 (DE3). A 50 ml culture of autoinduction medium (12 g l^−1^ tryptone, 24 g l^−1^ yeast extract, 100 mM potassium phosphate buffer pH 7.0, 0.5 g l^−1^ glucose, 2 g l^−1^ lactose, 100 μg ml^−1^ ampicillin) was inoculated by a few colonies and grown at 37 °C for 4 h before the temperature was reduced to 20 °C for another 20 h of incubation. For cells harboring the pKJE7 plasmid, the lactose was replaced by 0.5 g l^−1^ arabinose and protein expression was induced by a final concentration of 1 mM IPTG after 4 h. The cells were harvested and washed once with 100 mM potassium phosphate buffer at pH 7.0 before a final OD_600_ of 30 was established. Five hundred microliters of cell suspension was incubated for 2 h in 1.5 ml reaction tubes containing 2 mM substrate. GC-MS samples were prepared as described below to identify product formation.

### Protein production and purification

From a 100 ml preculture in LB medium, 4.5 l of autoinduction medium (12 g l^−1^ tryptone, 24 g l^−1^ yeast extract, 100 mM potassium phosphate buffer pH 7.0, 0.5 g l^−1^ glucose, 0.5 g l^−1^ arabinose, 100 μg ml^−1^ ampicillin) in a 5 l fermenter were inoculated to a starting OD_600_ of 0.05. The cultures were stirred at 300 rpm at 37 °C for 4 h before the temperature was reduced to 20 °C, and protein expression was induced by the addition of a final concentration of 1 mM IPTG upon reaching 20 °C. The cultures were incubated for another 20 h. During the whole time, 2 l min^−1^ air were supplied. The final OD_600_ ranged between 8 and 10. The cultures were harvested at 5000*g* for 20 min at 4 °C. Cell pellets were washed in 100 mM potassium phosphate buffer pH 7.0 and centrifuged again. The cell pellets were either directly used for cell lysis or stored at −20 °C for further use.

For lysis, cells were resuspended in buffer A (10 mM Tris–HCl pH 7.5, 500 mM NaCl) and lysed by sonication. The lysate was centrifuged at 20,000*g* for 1.5 h at 4 °C. The supernatant was collected, filtered through a 0.2 μm pore, and applied to an Äkta Start FPLC system from GE Healthcare. Affinity chromatography was performed using a 5 ml Ni-NTA-HisTrap column from GE Healthcare. The protein was eluted at 100% buffer B (10 mM Tris–HCl pH 7.5, 500 mM NaCl, 500 mM imidazole) while before a washing step of 20% buffer B in buffer A was performed. During the whole process, the absorbance at 280 nm was monitored to collect protein-containing fractions. The buffer was exchanged to 50 mM potassium phosphate buffer pH 7.5 by means of dialysis overnight at 8 °C. Afterward, the dialyzed proteins were concentrated by ultrafiltration (Amicon 10 KDa cut-off) and stored with a final concentration of 60% glycerol at −20 °C until further use.

### Size-exclusion chromatography

Analytical size-exclusion chromatography was carried out using a Superdex 200 Increase 10/300 Gl column (Cytiva) in 50 mM sodium phosphate at pH 7.2 containing 150 mM sodium chloride. A constant flow rate was applied using a Knauer Azura FPLC system. 150 µl of a 25 µM protein solution was injected to the column. As reference, the Cytiva high molecular weight gel filtration calibration kit was used. Detection was performed at 280 nm.

### Determination of protein concentration

The absorption of the covalently bound FAD cofactor was used to measure the amount of active protein in the solution. Measurement was carried out at 441 nm (ε = 14,200 M^−1^ cm^−1^) (6, 51). The total amount of protein was determined by Bradford assay using the RotiQuant reagent from Roth. Protein dilutions ranging from 10 to 1000 times were prepared in a volume of 200 μl containing a final concentration of 1x RotiQuant. The solution was incubated for 30 min in the dark before the absorption was measured at 595 nm. A standard curve with bovine serum albumin was used for protein quantification.

### Xylenol orange assay for enzyme activity measurements

To obtain kinetic data of the enzymes, a 96-well plate-based assay was used to photometrically determine the production of hydrogen peroxide, which is a stochiometric byproduct of VAOs. A variation of this assay was applied before (10); however, the sample points were increased from two to three to improve data quality. During the assay, triplicates of 100 μl, containing a final concentration of 50 mM potassium phosphate buffer pH 7.5, a varying amount of substrate, and 50 nM of the respective enzymes were incubated and shaken at 25 °C on a 96-well plate. The reactions were simultaneously started by the addition of enzymes. After 3, 6, and 9 min, 20 μl of each reaction were transferred onto a new 96-well plate, already containing 180 μl of detection solution (250 μM FeSO_4_, 25 mM H_2_SO_4_, 100 μM Xylenol-Orange). In the presence of hydrogen peroxide, the absorption of the dye shifts from 440 nm to 560 nm which was measured after incubation for 30 min. The amount of hydrogen peroxide was determined by a calibration curve, and the slope though all three measurement points was used to calculate the enzyme activity.

### pH stability assay

The activity assay was performed similar to the xylenol orange assay but using Briton-Robinson buffer (40 mM acetic acid, 40 mM phosphoric acid, 40 mM boric acid) in 0.5 pH steps, while vanillyl methyl ether at a final concentration of 2 mM was used for all reactions. For pH stability, the enzyme stock solution was incubated in Briton-Robinson buffer for 2 h before the reaction described in the xylenol orange assay was performed.

### Temperature stability assay

The activity assay was performed similar to the xylenol orange assay, but the enzyme aliquots were incubated at the respective temperature for 2 h before they were cooled on ice. Vanillyl methyl ether was used at a final concentration of 2 mM for all reactions.

### Total turnover assay

The total turnover was determined by measurement of the reaction product vanillin at 350 nm. The reaction was performed in triplicates of 200 μl in a 96-well plate, containing a final concentration of 50 mM potassium phosphate buffer pH 7.5, 10 mM of substrate, and 5 nM of the respective enzyme. The plate was incubated and shaken at 30 °C, and the absorption was measured in 15 min intervals. The turnover was calculated as the produced product molecules per active site.

### GC-MS measurements

To identify the formed products of the converted substrates, triplicates of 400 μl in 1.5 ml tubes of each substrate were used, containing 2 mM of substrate, 50 mM of potassium phosphate buffer pH 7.5, and 500 nM of the respective enzyme. The reactions were shaken at 25 °C and 750 rpm for 16 h. For sample preparation, 300 μl of the solution were extracted with the same volume of ethyl acetate by vortexing for 1 min. Phase separation was initiated by centrifugation for 5 min at 17,000*g* and room temperature. Afterward, the organic phase was dried over anhydrous MgSO_4_ and 100 μl were used for analysis. On a Shimadzu Nexis GC-MS 2030, a 5 °C min^−1^ gradient from 150 to 200 °C was applied followed by a 5 min hold at 200 °C using a FS-Supreme 5 ms column (length: 30 m, inner diameter: 0.25 mm, outer diameter: 0.36 mm). Compounds were identified by NIST 2017 library search.

## Data availability

All data will be available upon request.

## Supporting information

This article contains supporting information (52, 53, 54).

## Conflict of interest

The authors declare that they have no conflicts of interest with the contents of this article.
