# Supplementary material for: Vanillyl alcohol oxidase from Diplodia corticola: Residues Ala420 and Glu466 allow for efficient catalysis of syringyl derivatives
Source: J Biol Chem. 2023 Jun 8;299(7):104898. doi: 10.1016/j.jbc.2023.104898 (PMC10404669; doi:10.1016/j.jbc.2023.104898)
Supplement: Supporting information [file mmc1.pdf]

Supporting Information: Vanillyl alcohol oxidase from *Diplodia corticola*: Residues Ala420 and Glu466 allow for efficient catalysis of syringyl derivatives

Daniel Eggerichs[1], Nils Weindorf[1], Maria Laura Mascotti[2,3], Natalie Welzel[1], Marco W. Fraaije[2], Dirk Tischler[1]

[1] Microbial Biotechnology, Ruhr-University Bochum, Universitätsstraße 150, 44801 Bochum, Germany

[2] Molecular Enzymology, University of Groningen, Nijenborgh 4, 9747AG Groningen, The Netherlands

[3] IMIBIO-SL CONICET, Facultad de Química Bioquímica y Farmacia, Universidad Nacional de San Luis, Ejército de los Andes 950, D5700HHW San Luis, Argentina

Keywords: flavoprotein oxidase, lignin, ether cleavage, ancestral sequence reconstruction, enzyme engineering, biocatalysis

Table of content

UV/vis Spectrum of *Dc*VAO 3
Size exclusion chromatogram of *Dc*VAO 3
Temperature stability data of *Dc*VAO 4
pH stability of *Dc*VAO 4
Activity data for *Dc*VAO wt and variants and *Ps*VAO 5
Products detected by GC-MS for *Dc*VAO conversions 7
GC-MS chromatograms of *Dc*VAO hydroxylation/dehydrogenation reactions 10
Michaelis-Menten kinetics for *Dc*VAO and *Ps*VAO 12
Total turnover data for *Dc*VAO 14
Substrate orientation in the catalytic center 15
Autodocking results of *Dc*VAO 16
Ancestral sequence reconstruction data 17
Activity data for ancestral enzymes 18
Catalytic center of ancestral enzymes 20
Protein sequence 22
Primer 22
References 23

UV/vis Spectrum of *Dc*VAO

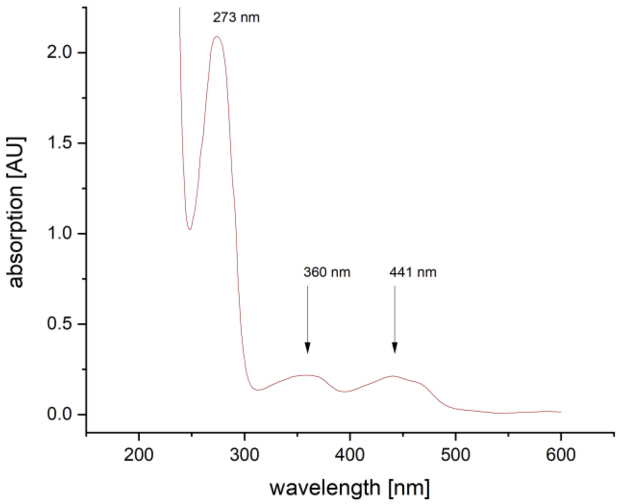

Figure S1: UV/vis spectrum of 50 µM *Dc*VAO in 50 mM potassium phosphate buffer at pH 7.5. Three peaks at 273, 360, 441 nm are visible which is characteristic for a covalent bound FAD cofactor.

Size exclusion chromatogram of *Dc*VAO

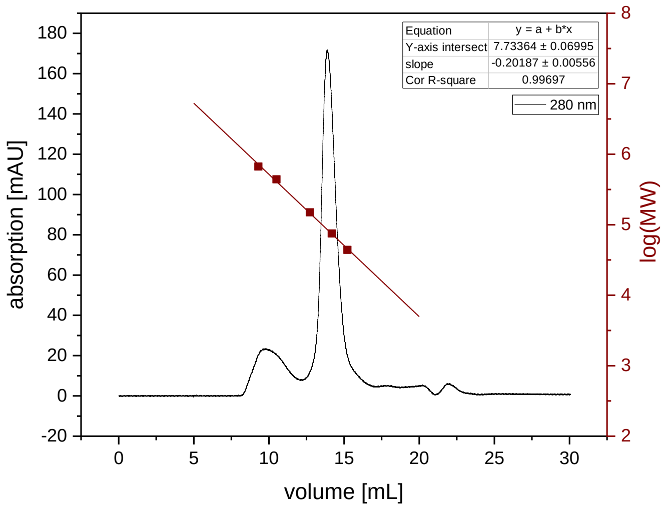

Figure S2. Elution chromatogram for *Dc*VAO from size exclusion column (black) and standard measurements (red) from the high molecular weight gel filtration calibration kit (Cytiva). The first peak of the elution profile corresponds to the size of an octamer (~504 kDa) while the main peak corresponds to the molecular weight of a dimer (~126 kDa). As reference proteins, ovalbumin (44 kDa), conalbumin (75 kDa), aldolase (150 kDa), ferritin (440 kDa) and thyroglobulin (670 kDa) were used. The elution volume is shown against the logarithm of the molecular weight.

Temperature stability data of *Dc*VAO

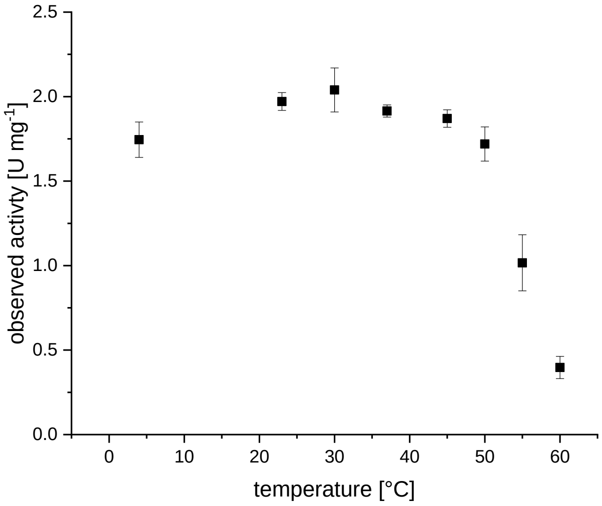

Figure S3: Residual activity on vanillyl methyl ether (**13**) of *Dc*VAO after incubation for 2 h at the indicated temperature in 50 mM potassium phosphate buffer at pH 7.5. The enzyme is stable up to 50°C and the melting point can be estimated around 55°C. All reactions were performed as triplicates and the standard deviation is shown as error bars.

pH stability of *Dc*VAO

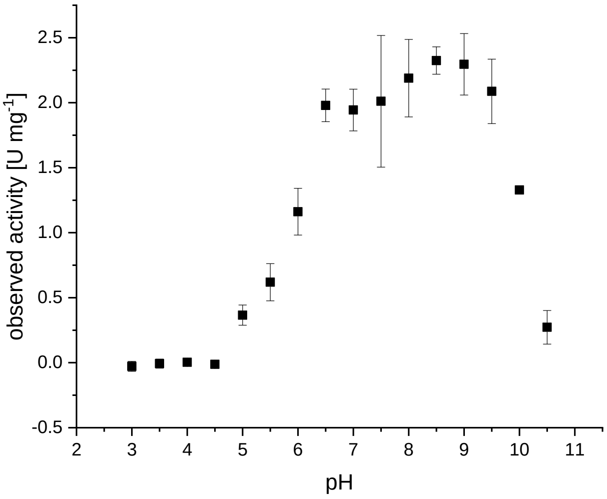

Figure S4: Initial activity of *Dc*VAO on vanillyl methyl ether (**13**) in Briton-Robinson buffer at the indicated pH value. The enzyme exhibits maximal activity between a pH range of 6.5 and 9.5. All reactions were performed as triplicates and the standard deviation is shown as error bars.

Activity data for *Dc*VAO wt and variants and *Ps*VAO

Table S1: Activity data of *Dc*VAO wt, respective variants and *Ps*VAO as reference. All measurements were performed in the first nine minutes of reaction time according to the xylenol orange assay. The reactions were performed in 50 mM potassium phosphate buffer at pH 7.5 at 25°C. All activity data is given in U mg^{-1}. For substrates, R_3 represents the reactive group where the benzylic carbon atom for hydride abstraction is located. R_1 and R_2 in *ortho* position to the electron donating group X have a steric effect. For most substrates, X is an OH group, but aniline and thiol derivatives were tested as well.

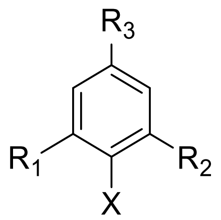

Nr	Name	X	R1	R2	R3	*Dc*VAO	*Dc*VAO E466C	*Dc*VAO E466L	*Ps*VAO
1	Chavicol	OH	H	H	Allyl	9.5 ± 0.5	1.0 ± 0.4	0.9 ± 0.1	9.3 ± 0.1
2	Eugenol	OH	OMe	H	Allyl	9.9 ± 0.1	0.5 ± 0.03	0.7 ± 0.1	17.2 ± 0.2
3	4-allyl-2,6-dimethoxy-phenol	OH	OMe	OMe	Allyl	3.5 ± 0.02	0.2 ± 0.05	0.2 ± 0.03	0.2 ± 0.1
4	4-Hydroxybenzyl alcohol	OH	H	H	CH2OH	1.2 ± 0.04	0.5 ± 0.2	0.2 ± 0.07	<0.1
5	Vanillyl alcohol	OH	OMe	H	CH2OH	3.4 ± 0.1	1.7 ± 0.3	0.4 ± 0.07	3.7 ± 0.5
6	4-(hydroxymethyl)-2,6-dimethoxyphenol	OH	OMe	OMe	CH2OH	6.3 ± 0.5	1.0 ± 0.07	0.5 ± 0.05	<0.1
7	3,4-Dihydroxybenzyl alcohol	OH	OH	H	CH2OH	0.5 ± 0.1	0.7 ± 0.1	0.5 ± 0.05	2.0 ± 0.7
8	3-Bromo-4-hydroxybenzyl alcohol	OH	Br	H	CH2OH	0.1 ± 0.1	n.d.	n.d.	0.2 ± 0.1
9	4-(1-Hydroxyethyl)phenol	OH	H	H	CHOHCH3	3.6 ± 0.3	1.0 ± 0.2	0.5 ± 0.04	8.2 ± 0.3
10	4-(1-Aminoethyl)phenol	OH	H	H	CHNH2CH3	<0.1	0.1 ± 0.03	n.d.	0.2 ± 0.03
11	Vanillylamine	OH	OMe	H	CH2NH2	n.d.	n.d.	n.d.	<0.1
12	4-Aminobenzylalkohol	NH2	H	H	CH2OH	<0.1	n.d.	n.m.	0.1 ± 0.03
13	Methyl vanillyl ether	OH	OMe	H	MeOMe	1.5 ± 0.1	0.7 ± 0.04	0.3 ± 0.01	6.9 ± 0.1
14	Ethyl vanillyl ether	OH	OMe	H	MeOEt	4.0 ± 0.1	2.5 ± 0.1	0.8 ± 0.05	1.8 ± 0.1
15	Butyl vanillyl ether	OH	OMe	H	MeOBu	1.5 ± 0.2	1.8 ± 0.02	<0.1	<0.1
16	4-Methoxymethyl-phenol	OH	H	H	MeOMe	0.6 ± 0.1	0.3 ± 0.2	n.d.	3.0 ± 0.1
17	4-(2-Methoxyethyl)phenol	OH	H	H	EtOMe	<0.1	n.d.	n.d.	0.3 ± 0.1
18	4-Ethylphenol	OH	H	H	Ethyl	2.0 ± 0.1	0.2 ± 0.03	0.2 ± 0.01	3.8 ± 0.4
19	4-Ethylcatechol	OH	OH	H	Ethyl	0.7 ± 0.2	n.d.	0.5 ± 0.2	1.2 ± 0.3
20	4-Ethylguaiacol	OH	OMe	H	Ethyl	7.6 ± 0.3	1.0 ± 0.2	0.7 ± 0.09	7.4 ± 0.8
21	4-Ethylaniline	NH2	H	H	Ethyl	n.d.	n.d.	n.m.	<0.1
22	4-Ethylthiophenol	SH	H	H	Ethyl	n.d.	n.d.	n.m.	<0.1
23	para-Cresol	OH	H	H	Methyl	<0.1	<0.1	n.d.	<0.1
24	4-Methylcatechol	OH	OH	H	Methyl	<0.1	n.d.	0.8 ± 0.1	2.6 ± 0.3
25	2-Methoxy-4-Methyl-phenol	OH	OMe	H	Methyl	<0.1	n.d.	n.d.	<0.1

Table S1 continued

Nr	Name	X	R1	R2	R3	DcVAO	DcVAO E466C	DcVAO E466L	PsVAO
26	2-Amino-4-methyl-phenol	OH	NH2	H	Methyl	n.d.	n.d.	0.2 ± 0.03	n.d.
27	2-Chloro-p-cresol	OH	Cl	H	Methyl	<0.1	n.d.	n.d.	<0.1
28	2-Brom-4-methyl-phenol	OH	Br	H	Methyl	<0.1	n.d.	<0.1	<0.1
29	p-Toluidine	NH2	H	H	Methyl	<0.1	n.d.	n.m.	<0.1
30	p-Toluenethiol	SH	H	H	Methyl	n.d.	n.d.	n.m.	<0.1
31	4-Propylphenol	OH	H	H	Propyl	2.8 ± 0.2	<0.1	0.1 ± 0.08	4.9 ± 0.04
32	4-Butylphenol	OH	H	H	Butyl	0.7 ± 0.1	<0.1	<0.1	1.4 ± 0.01
33	4-Isopropylphenol	OH	H	H	iso-Propyl	0.4 ± 0.1	<0.1	n.d.	1.5 ± 0.04
34	4-sec-Butylphenol	OH	H	H	sec-Butyl	0.2 ± 0.1	n.d.	n.d.	0.4 ± 0.03
35	4-Cyclopentylphenol	OH	H	H	Cyclopenytl	3.5 ± 0.3	0.8 ± 0.2	0.3 ± 0.04	8.5 ± 0.4
36	4-Cyclohexylphenol	OH	H	H	Cyclohexyl	0.1 ± 0.1	n.d.	n.d.	0.6 ± 0.1
37	5-Hydroxyindan	OH	H	H		1.1 ± 0.03	0.9 ± 0.3	0.2 ± 0.06	0.4 ± 0.01
38	5,6,7,8-Tetrahydro-2-naphtol	OH	H	H		<0.1	0.6 ± 0.2	<0.1	0.6 ± 0.02

n.d. = not detected, n.m. = not measured

Products detected by GC-MS for *Dc*VAO conversions

Table S2: Chemical structure of *Dc*VAO reaction products identified by NIST2017 library search. All compounds were measured by a GC-MS after 16 h reaction time. The reactions were performed in 50 mM potassium phosphate buffer at pH 7.5 at 25°C. For all enzyme variants, the same products were observed.

Nr	Name	Substrate	Main product	Side product(s)
**1**	Chavicol	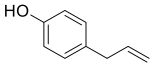	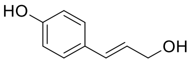	n.d.
**2**	Eugenol	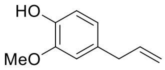	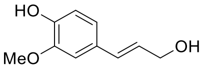	n.d.
**3**	4-allyl-2,6-dimethoxy-phenol	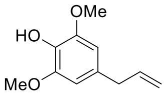	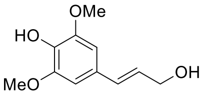	n.d.
**4**	4-Hydroxybenzyl alcohol	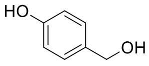	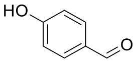	n.d.
**5**	Vanillyl alcohol	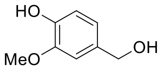	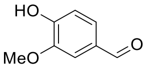	n.d.
**6**	4-(hydroxymethyl)-2,6-dimethoxyphenol	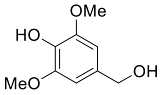	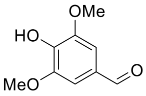	n.d.
**7**	3,4-Dihydroxybenzyl alcohol	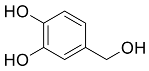	n.d.	n.d.
**8**	3-Bromo-4-hydroxybenzyl alcohol	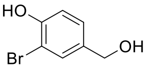	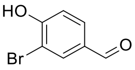	n.d.
**9**	4-(1-Hydroxyethyl)phenol	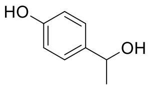	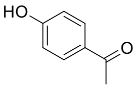	n.d.
**10**	4-(1-Aminoethyl)phenol	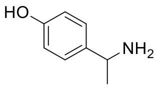	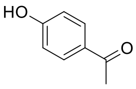	n.d.
**11**	Vanillylamine	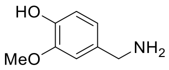	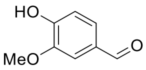	n.d.
**12**	4-Aminobenzylalkohol	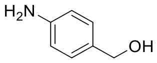	n.d.	n.d.
**13**	Methyl vanillyl ether	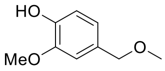	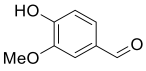	n.d.
**14**	Ethyl vanillyl ether	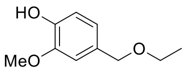	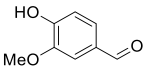	n.d.
**15**	Butyl vanillyl ether	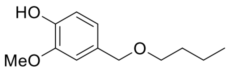	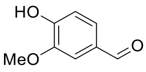	n.d.
**16**	4-Methoxymethylphenol	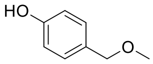	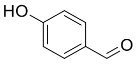	n.d.
**17**	4-(2-Methoxyethyl)phenol	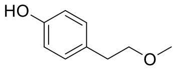	n.d.	n.d.

Table S2 continued

Nr	Name	Substrate	Main product	Side product(s)
**18**	4-Ethylphenol	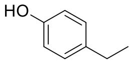	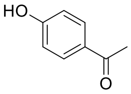	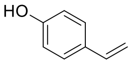 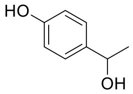
**19**	4-Ethylcatechol	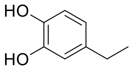	n.d.	n.d.
**20**	4-Ethylguaiacol*	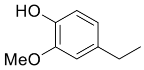	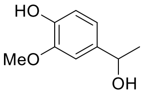	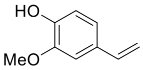 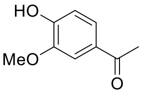
**21**	4-Ethylaniline	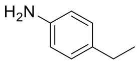	n.d.	n.d.
**22**	4-Ethylthiophenol	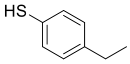	n.d.	n.d.
**23**	*para*-Cresol	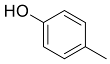	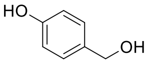	n.d.
**24**	4-Methylcatechol	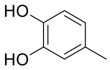	n.d.	n.d.
**25**	2-Methoxy-4-Methyl-phenol	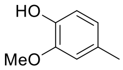	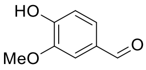	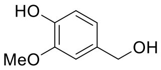
**26**	2-Amino-4-methyl-phenol	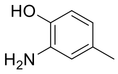	n.d.	n.d.
**27**	2-Chloro-p-cresol	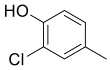	n.d.	n.d.
**28**	2-Brom-4-methyl-phenol	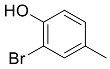	n.d.	n.d.
**29**	p-Toluidine	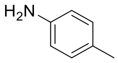	n.d.	n.d.
**30**	p-Toluenethiol	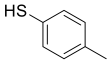	n.d.	n.d.
**31**	4-Propylphenol	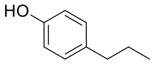	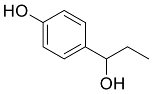	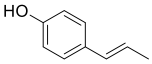 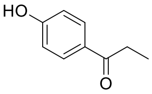
**32**	4-Butylphenol	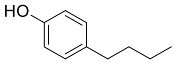	n.d.	n.d.
**33**	4-Isopropylphenol	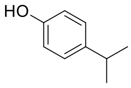	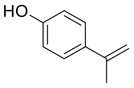	n.d.
**34**	4-sec-Butylphenol	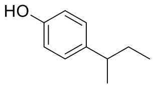	n.d.	n.d.
**35**	4-Cyclopen-tylphenol*	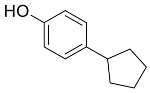	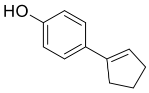	n.d.

Table S2 continued

Nr	Name	Substrate	Main product	Side product(s)
**36**	4-Cyclohexylphenol	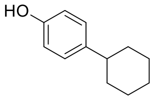	n.d.	n.d.
**37**	5-Hydroxyindan*	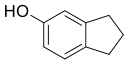	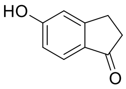	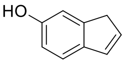
**38**	5,6,7,8-Tetrahydro-2-naphtol	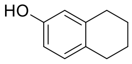	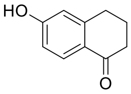	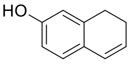

*see Figure S5 to S8 for further details, n.d. = not detected

GC-MS chromatograms of *Dc*VAO hydroxylation/dehydrogenation reactions

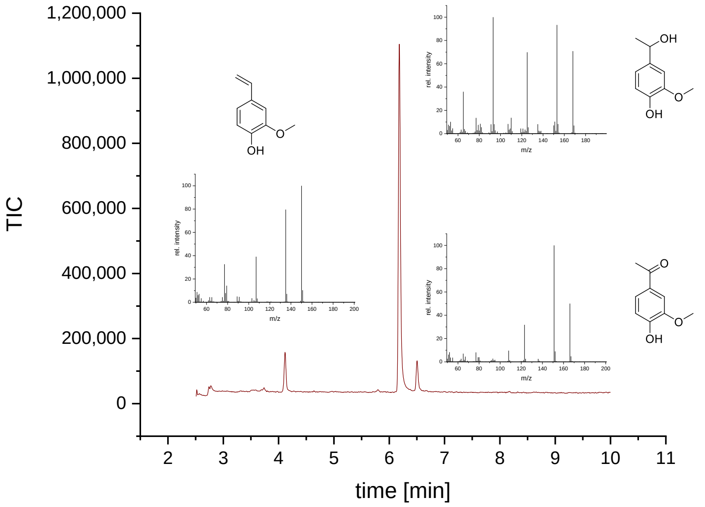

Figure S5: Total ion chromatogram of a 16 h reaction of ethyl guaiacol (**37**) by *Dc*VAO. No remaining substrate could be detected while the hydroxylated product (ret. time 6.2 min) was found predominantly. Smaller amounts of the dehydrogenated product can be found at a ret. Time of 4.2 min. At 6.5 minutes the dehydrogenation product of the hydroxylated can be seen in small amounts.

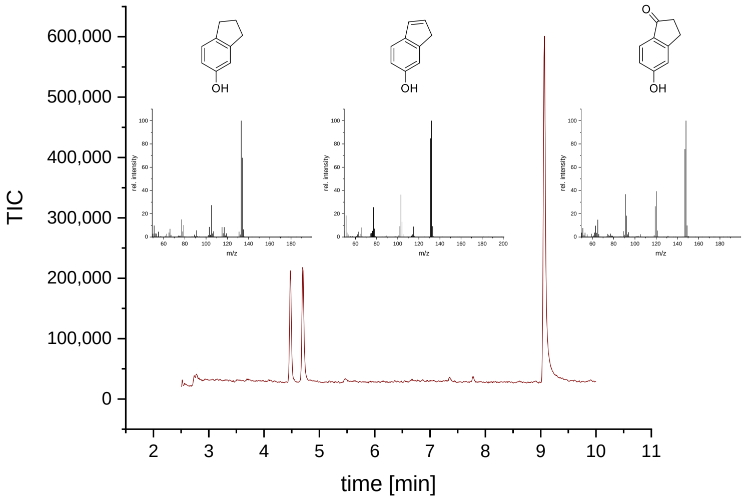

Figure S6: Total ion chromatogram of a 16 h conversion of 5-hydroxyindan (**37**) by *Dc*VAO. Remaining substrate was detected at a retention time of 4.5 min. The dehydrogenated and the respective ketone were observed while later is the dominating peak (ret. time 4.7 and 9.1 min respectively). The hydroxylated product was not observed. It is likely that *Dc*VAO exhibits higher activity towards this compound than for 5-hydroxyindan itself shifting the reaction towards ketone formation.

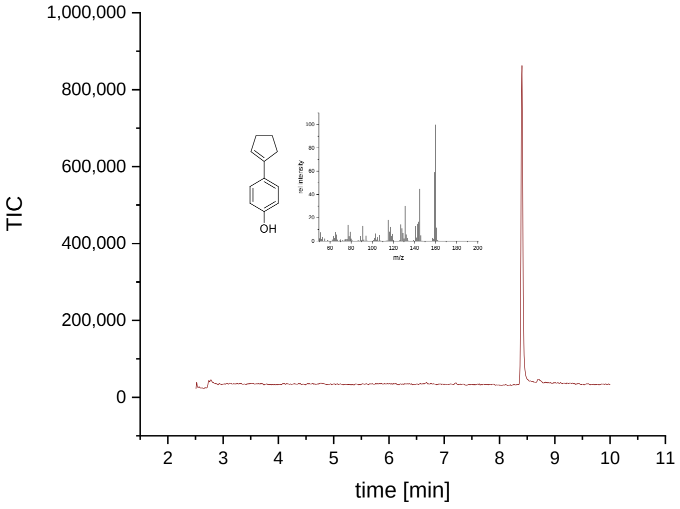

Figure S7: Total ion chromatogram of a 16 h conversion of 4-cyclopentyl phenol (**35**) by *Dc*VAO. No substrate could be observed while the only detected compound was the dehydrogenated product (ret. time 8.4 min).

Michaelis-Menten kinetics for *Dc*VAO and *Ps*VAO

For most kinetics, the classical Michaelis-Menten behavior was observed. Thus, the Michaelis-Menten equation was applied for fitting where v_{max} represents the maximal activity and K_M the Michaelis constant.

$$v = \frac{v_{max}[S]}{K_M + [S]} \tag{1}$$

For eugenol, additional effects were observed. For fitting of cooperative effects, the Hill equation (1) was used where the additional parameter n describes the degree of cooperativity.

$$v = \frac{v_{max}}{1 + \frac{K^n}{[S]^n}} \tag{2}$$

For *Ps*VAO, we further observed substrate inhibition which was described according to Haldane.(2) Here, K_i represents the inhibition constant.

$$v = \frac{v_{max}}{1 + \frac{K}{[S]} + \frac{[S]}{K_i}} \tag{3}$$

As for *Ps*VAO cooperativity and inhibition were observed at the same time, a model according to LiCata was applied which combines Hill and Haldane equation.(3) For simplicity, we assume that there is a single inhibition side per enzyme and the inhibition complex is catalytically inactive.

$$v = \frac{v_{max}}{1 + \frac{K^n}{[S]^n} + \frac{[S]}{K_i}} \tag{4}$$

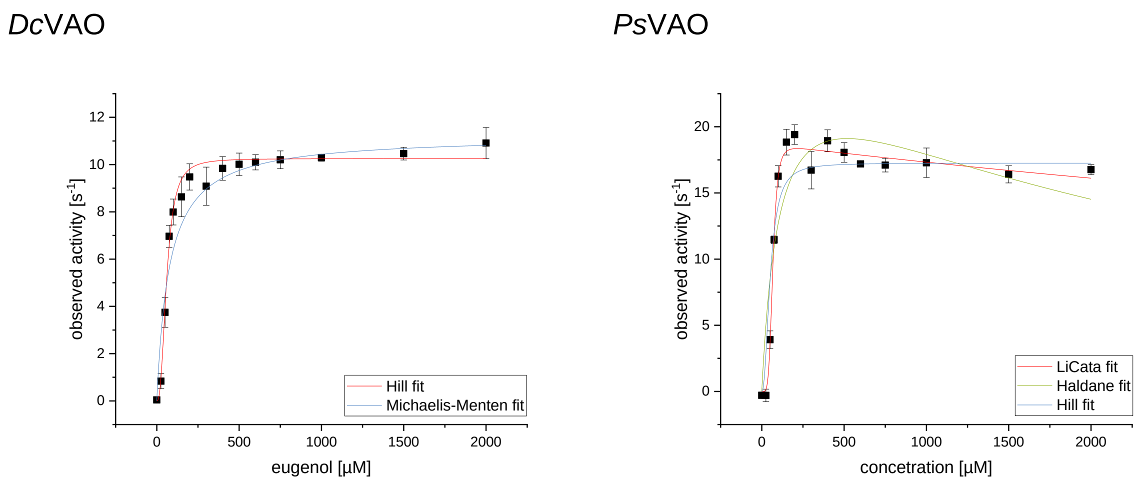

Figure S8: Michaelis-Menten kinetic on eugenol (**2**) for *Dc*VAO (left) and *Ps*VAO (right). The reaction was performed in 50 mM potassium phosphate buffer at pH 7.5. Cooperativity was observed for both enzymes while for *Ps*VAO also substrate inhibition was observed. Thus, the Hill equation was applied to fit the data points for *Dc*VAO. The alternative fit without cooperativity is shown for comparison (left). The data points for PsVAO were fitted according to a combined model of cooperativity and substrate inhibition by LiCata *et. al*. For comparison, fits with the Haldane equation for substrate inhibition and the Hill equation for cooperativity are shown (right). For kinetic parameters see Table 1 in the main text. Standard deviation of a triplicate measurement is shown as error bars.

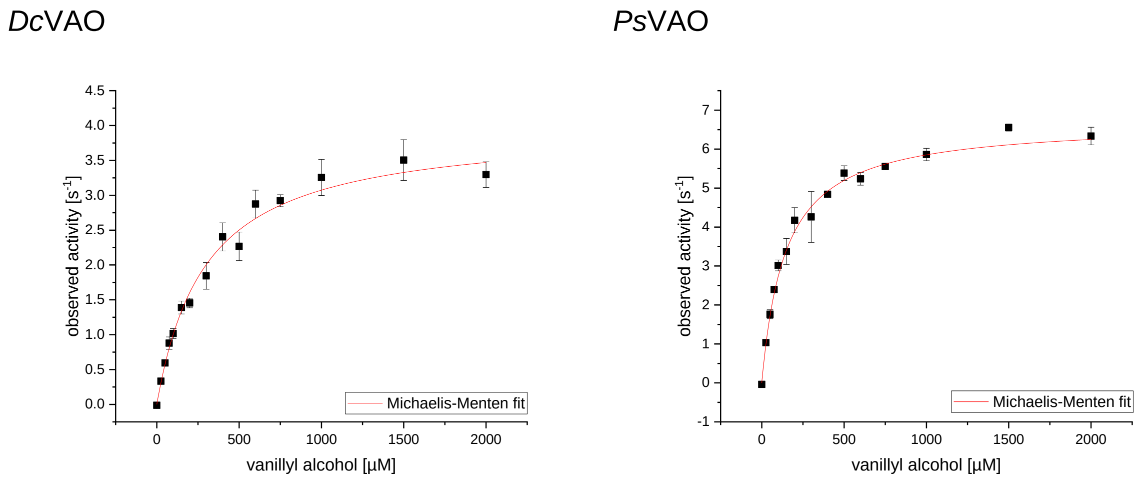

Figure S9: Michaelis-Menten kinetic on vanillyl alcohol (**5**) for *Dc*VAO (left) and *Ps*VAO (right). The reaction was performed in 50 mM potassium phosphate buffer at pH 7.5. Data points were fitted according to the Michaelis-Menten equation. For kinetic parameters see Table 1 in the main text. Standard deviation of a triplicate measurement is shown as error bars.

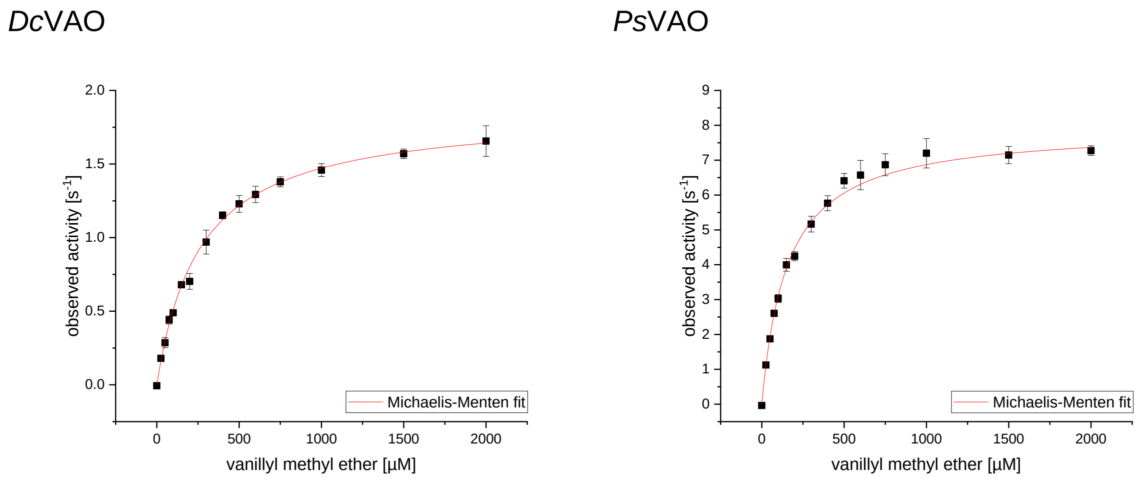

Figure S10: Michaelis-Menten kinetic on vanillyl methyl ether (**13**) for *Dc*VAO (left) and *Ps*VAO (right). The reaction was performed in 50 mM potassium phosphate buffer at pH 7.5. Data points were fitted according to the Michaelis-Menten equation. For kinetic parameters see Table 1 in the main text. Standard deviation of a triplicate measurement is shown as error bars.

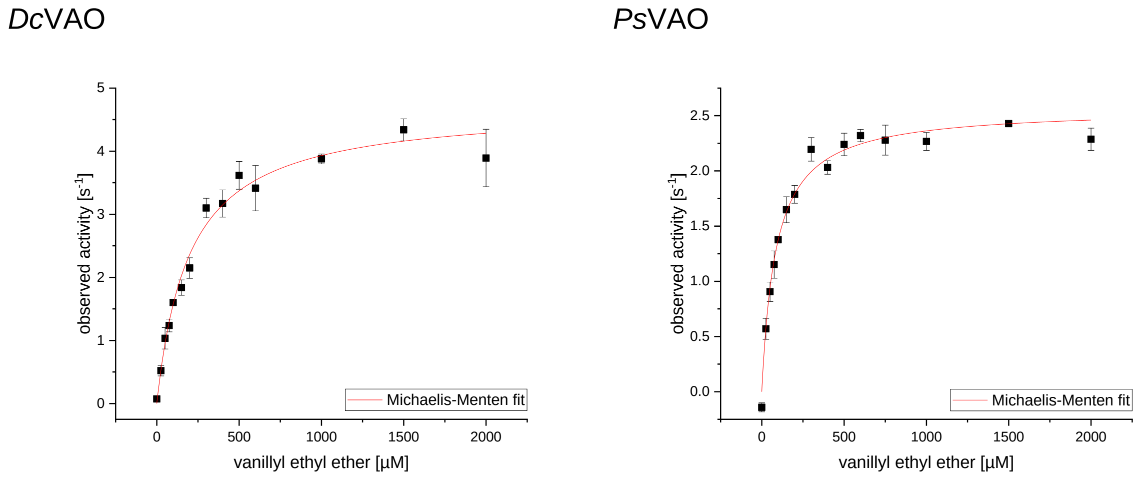

Figure S11: Michaelis-Menten kinetic on vanillyl ethyl ether (**14**) for *Dc*VAO (left) and *Ps*VAO (right). The reaction was performed in 50 mM potassium phosphate buffer at pH 7.5. Data points were fitted according to the Michaelis-Menten equation. For kinetic parameters see Table 1 in the main text. Standard deviation of a triplicate measurement is shown as error bars.

*Dc*VAO

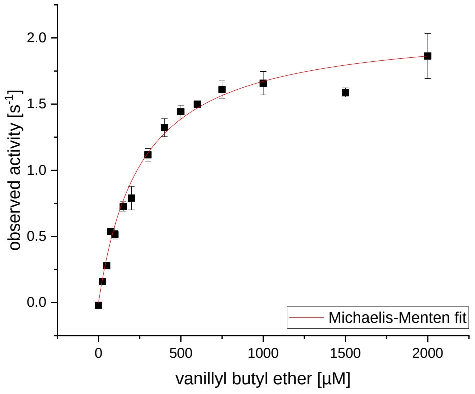

Figure S12: Michaelis-Menten kinetic on vanillyl butyl ether (**15**) for *Dc*VAO (left). No activity was observed for *Ps*VAO. The reaction was performed in 50 mM potassium phosphate buffer at pH 7.5. Data points were fitted according to the Michaelis-Menten equation. For kinetic parameters see Table 1 in the main text. Standard deviation of a triplicate measurement is shown as error bars.

Total turnover data for *Dc*VAO

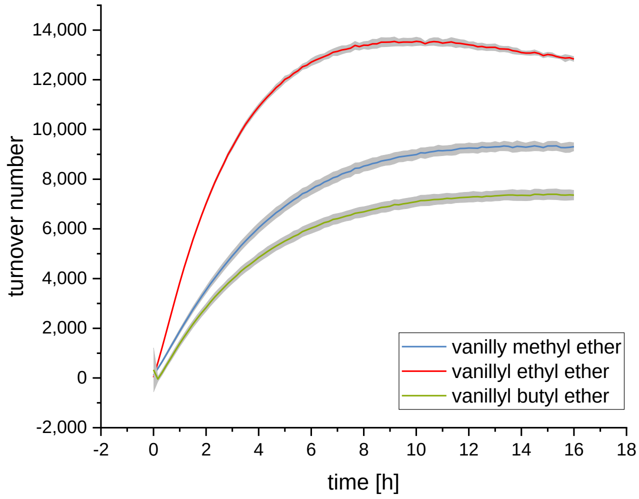

Figure S13: Turnover number for *Dc*VAO on indicated ether compounds. The absorption of the product vanillin was measured at 350 nm for quantification. The reaction was performed in triplicates in 50 mM potassium phosphate buffer at pH 7.5. The grey area represents the standard error of the replicates.

Substrate orientation in the catalytic center

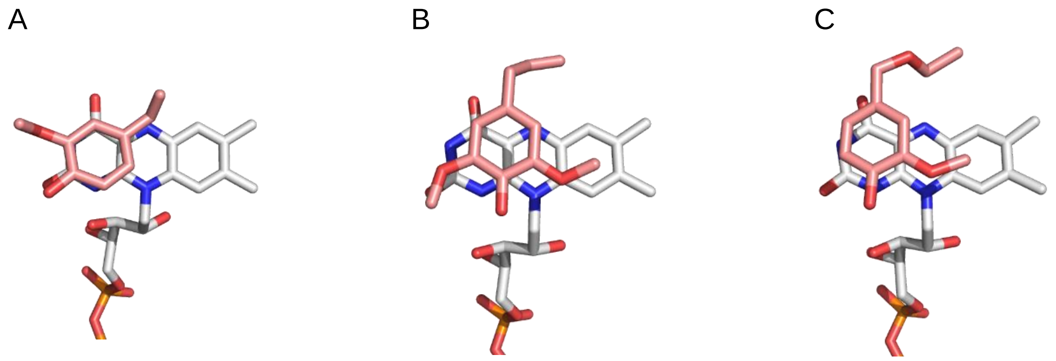

Figure S14: Comparison of the substrate binding modes in *Ps*VAO bound with 4-ethylguiacol (A, PDB 2VAO), in *Dc*VAO docked with 4-allyl-2,6-dimethoxy-phenol (B) and vanillyl ethyl ether (C). Due to Ala instead of Phe in position 420 (*Dc*VAO numbering) substrate binds in a rotated position which is similar to *Rj*EUGO. This position allows for the accommodation of dimethoxylated compounds.

Autodocking results of *Dc*VAO

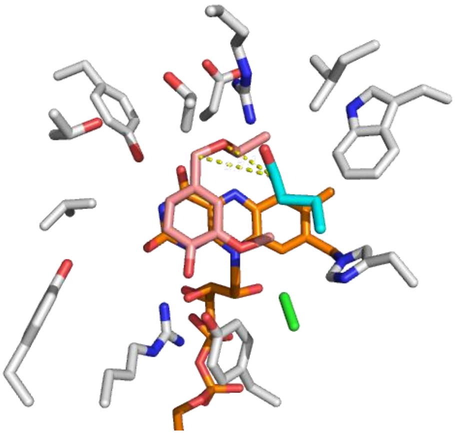

Figure S15: Catalytic center of the homology model of *Dc*VAO with vanillyl ethyl ether (**14**) bound after energy minimization. Glu466 is in proximity to the benzylic position (O_{Glu}-C_{α}: 4.7 Å) and the oxygen in beta position (O_{Glu}-O_{β}: 4.1 Å). Glu470 is highlighted in teal while A420 is depicted in green. The substrate is colored light red, and the FAD cofactor is colored orange. The shortest distances between O_{Glu} and the substrate are depicted as yellow, dashed lines.

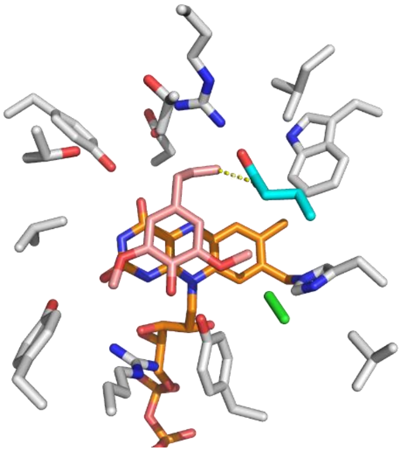

Figure S16: Catalytic center of the homology model of *Dc*VAO with 4-allyl-2,6-dimethoxy-phenol (**3**) bound after energy minimization. Ala420 leaves the space required for binding a substrate with two *o*-methoxy groups. The positioning of the substrate brings Glu466 in proximity to the gamma position (O_{Glu}-C_{γ}: 3.7 Å). Glu470 is highlighted in teal while A420 is depicted in green. The substrate is colored light red, and the FAD cofactor is colored orange. The shortest distances between O_{Glu} and the substrate are depicted as yellow, dashed lines.

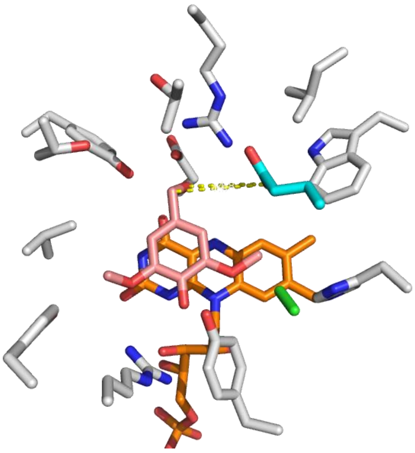

Figure S17: Catalytic center of the homology model of *Dc*VAO with 4-(hydroxymethyl)-2,6-dimethoxyphenol (**6**) bound after energy minimization. Ala420 leaves the space required for binding a substrate with two *o*-methoxy groups. Glu466 is in proximity to the benzylic position (O_{Glu}-C_{α}: 4.8 Å) and the oxygen in beta position (O_{Glu}-O_{β}: 4.8 Å). Glu470 is highlighted in teal while A420 is depicted in green. The substrate is colored light red, and the FAD cofactor is colored orange. The shortest distances between O_{Glu} and the substrate are depicted as yellow, dashed lines.

Ancestral sequence reconstruction data

Sequences of targeted nodes are presented along with the graphs showing the accuracy of reconstruction per site.

>AncVAO
MSSTNNVPSKAARESANQRPLVLPPGVSAEAFNEALEELRKVVGDEN-
VHVHTNDEPLEDGHYMNLPKTHDPYHVLDQDEFVASAVVCPGSTEEVQAIVRLANKYGIPLWPISIGRNLGYGGAAPRLRGSV
VLDLGKRMNKILEVNEKNAYCLVEPGVTYFDLYDYLQKHGLRDKL-
WIDCPDLGGGSVVGNALDRGVGYTPYGDHFMMHCGMEVVLPNGEVIRTGMGALPDPKSPEKEGARPEDQPGNNTWQLFPYGFG
PYPDGIFTQSNFGIVTKMGIWLMPNPGGYQSYMITFPREEDLEQIVEIIR-
PLRINMVIQNVPTIRNVLLDAAVMGSKSDYYDGDGPLPDEEIEKIAKKLNIGRWNFYGALYGPEPMRDAQWEVIKEAFSQIPG
ARFYFPEDRKENSVLHTRAKTMAGIPNLTELNWLNWRPNGAHLGF-
SPISPATGEDAMKQYQMVKKRCREYGFDYIGTFVVGWREMHHIVCLVFDRTDPEQRKRAHRCIRELIDDAAAQGYGEYRTHLA
FMDQVAGTYNWNNNALMKFNEKIKDALDPNGILAPGKSGIWPKRLRGRGWEL

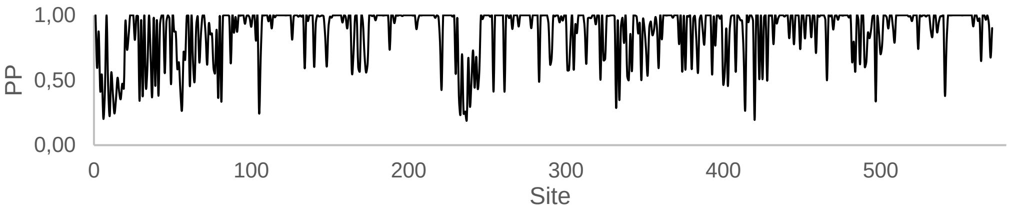

>AncPsVAO
MSPTNSVPSSSTDDGASQKPLVLPPGVSQETFNEFISEVREVVGDEN-
VTVITSKDQIDDGSYMNPTYTHDPHHVLEQDYFLASAVVAPRNVADVQAIVRLANKFQVPLWPISIGRNSGYGGA
APRVRGSVVLNMGKHMNKILEVNVEGAYCLVEPGVTFHDLHEYLVEHNLRDKL-
WIDVPDLGGGSVLGNTLERGVGYTPYGDHWMMHCGMEVVLPNGELIRTGMGALPDPKSPETEGARPEDQPWNKTA
QLFPYGFGPYVDGLFTQSNLGIVTKMGMWLMPNPGGYQSYLITIPRDEDLKQAVDIIR-
PLRLNMVLQNVPTIRHILLDAAVMGSKSDYTSSNGPLTDEELDAIAKKLNLGRWNFYGALYGPEPIRNAMWAVIK
EAFSAIPGAKFYFPEDTKENSVLHTRHKTMQGIPTFDELKWVDWRPNGAHLFFSPIAK-
VTGDDAMAQYAITKKRCREAGLDFIGTFTVGMREMHHIVCIVFDRKDPEQKKKAHWLIKTLIDDCAAQGWGEYRT
HLAVMDQIMGTYNWNNNALLKFNETIKNAVDPNGILAPGKSGVWPKQYNKREWKL

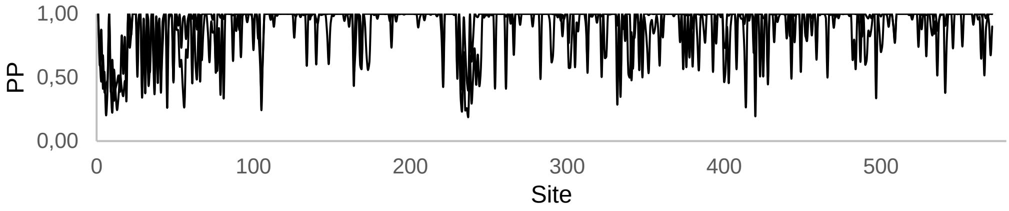

>AncDcVAO
MSSTSNVPTSAANDPLALPPGTSQETFSEFISEVREVVGEEN-
VTVITSKDELDDGSYMNPPKTHDPHHVLDQDYFVASAVVCPRSVPEVQAIVRLANEFQIPLWPISIGRNSGYGGA
APRLRGSVVLDMGKHMNRVLEVNVDGAYAVVEPGVTFADLHEYLVEHNLRDKL-
WIDVPDLGGGSVMGNTLERGVGYTPYGDHWMMHCGMEVVLPSGELIRTGMGALPDPKSPSKSGARPDEQPGNKCW
QLFPYGFGPYNDGLFSQSNLGIVTKMGIWLMPNPGGYQSYLITLPRDEDLHQAVDIIR-
PLRLQMVLQNVPTLRHILLDAAVMGSKSDYTSSNGPLNDEELDAIAKKLNLGRWNFYGALYGPEPIRNAMWSLIK
EAFSAIPGAKFFFPEDTKEPNSVLHTRHKTLQGIPTFDELKWVDWRPNGAHLFFSPISKIS-
GDDAMLQYSITKKRCREAGLDFIGTFTVGMREMHHIVCIVFDRKDPESKKKAHWLIKTLIDDCAAHGWGEYRTHL
ALMDQIAGTYNWNNNALMKFNETIKNALDPKGILAPGKNGVWPKSYDRRAWKLPGA

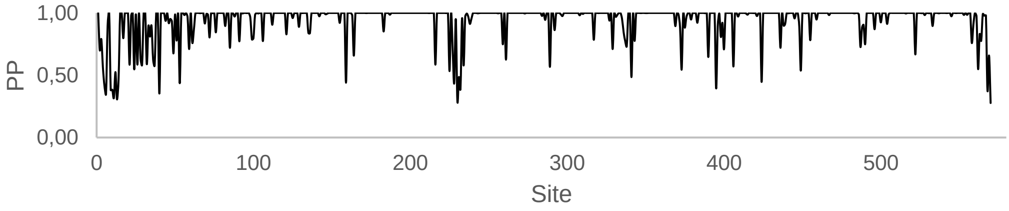

Activity data for ancestral enzymes

Table S3: Activity data of ancestral enzymes. All measurements were performed in the first nine minutes of reaction time according to the xylenol orange assay. The reactions were performed in 50 mM potassium phosphate buffer at pH 7.5 at 25°C. All activity data is given in U mg^{-1}. For substrates, R_3 represents the reactive group where the benzylic carbon atom for hydride abstraction is located. R_1 and R_2 in *ortho* position to the electron donating group X have a steric effect. For most substrates, X is an OH group, but aniline and thiol derivatives were tested as well.

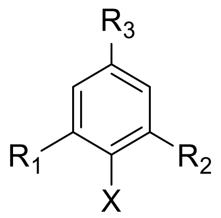

Nr	Group*	Name	X	R1	R2	R3	Ancestral VAO	Ancestral *Dc*VAO	Ancestral *Ps*VAO
1	A	Chavicol	OH	H	H	Allyl	0.95 ± 0.21	n.d.	0.96 ± 0.02
2	A	Eugenol	OH	OMe	H	Allyl	1.00 ± 0.09	0.06 ± 0.03	1.01 ± 0.02
3	A	4-allyl-2,6-dimethoxy-phenol	OH	OMe	OMe	Allyl	0.09 ± 0.04	0.08 ± 0.02	0.13 ± 0.02
4	B	4-Hydroxybenzyl alco-hol	OH	H	H	CH2OH	0.31 ± 0.06	n.d.	0.30 ± 0.06
5	B	Vanillyl alcohol	OH	OMe	H	CH2OH	1.32 ± 0.11	0.22 ± 0.03	0.68 ± 0.16
6	B	4-(hydroxymethyl)-2,6-dimethoxyphenol	OH	OMe	OMe	CH2OH	n.d.	0.22 ± 0.12	n.d.
7	B	3,4-Dihydroxybenzyl alcohol	OH	OH	H	CH2OH	0.97 ± 0.07	0.31 ± 0.04	0.44 ± 0.05
8	B	3-Bromo-4-hy-droxybenzyl alcohol	OH	Br	H	CH2OH	n.d.	0.16 ± 0.04	n.d.
9	B	4-(1-Hydroxy-ethyl)phenol	OH	H	H	CHOHCH3	0.70 ± 0.09	n.d.	1.04 ± 0.02
10	C	4-(1-Aminoethyl)phe-nol	OH	H	H	CHNH2CH3	n.d.	0.15 ± 0.07	0.09 ± 0.04
11	C	Vanillylamine	OH	OMe	H	CH2NH2	n.d.	n.d.	n.d.
12	-	4-Aminobenzylalkohol	NH2	H	H	CH2OH	n.d.	0.02 ± 0.01	n.d.
13	D	Methyl vanillyl ether	OH	OMe	H	MeOMe	n.m.	n.m.	n.m.
14	D	Ethyl vanillyl ether	OH	OMe	H	MeOEt	n.d.	0.23 ± 0.16	0.19 ± 0.04
15	D	Butyl vanillyl ether	OH	OMe	H	MeOBu	n.d.	0.29 ± 0.11	n.d.
16	D	4-Methoxymethyl-phe-nol	OH	H	H	MeOMe	n.d.	n.d.	0.15 ± 0.03
17	-	4-(2-Me-thoxyethyl)phenol	OH	H	H	EtOMe	n.d.	n.d.	n.d.
18	F	4-Ethylphenol	OH	H	H	Ethyl	n.d.	n.d.	0.15 ± 0.04
19	F	4-Ethylcatechol	OH	OH	H	Ethyl	0.33 ± 0.04	0.10 ± 0.05	n.d.
20	F	4-Ethylguaiacol	OH	OMe	H	Ethyl	n.d.	n.d.	0.45 ± 0.07
21	F	4-Ethylaniline	NH2	H	H	Ethyl	n.d.	n.d.	n.d.
22	F	4-Ethylthiophenol	SH	H	H	Ethyl	n.d.	n.d.	n.d.
23	E	para-Cresol	OH	H	H	Methyl	n.d.	n.d.	0.03 ± 0.01
24	E	4-Methylcatechol	OH	OH	H	Methyl	0.44 ± 0.03	0.80 ± 0.06	0.36 ± 0.11
25	E	2-Methoxy-4-Methyl-phenol	OH	OMe	H	Methyl	n.d.	n.d.	n.d.
26	E	2-Amino-4-methyl-phenol	OH	NH2	H	Methyl	n.d.	n.d.	n.d.

Table S2 continued

Nr		Name	X	R1	R2	R3	Ancestral VAO	Ancestral *Dc*VAO	Ancestral *Ps*VAO
27	E	2-Chloro-p-cresol	OH	Cl	H	Methyl	n.d.	n.d.	n.d.
28	E	2-Brom-4-methyl-phenol	OH	Br	H	Methyl	n.d.	n.d.	n.d.
29	E	p-Toluidine	NH2	H	H	Methyl	n.d.	n.d.	n.d.
30	E	p-Toluenethiol	SH	H	H	Methyl	n.d.	n.d.	n.d.
31	G	4-Propylphenol	OH	H	H	Propyl	n.d.	n.d.	0.22 ± 0.05
32	G	4-Butylphenol	OH	H	H	Butyl	n.d.	n.d.	n.d.
33	G	4-Isopropylphenol	OH	H	H	iso-Propyl	n.d.	n.d.	0.13 ± 0.04
34	G	4-sec-Butylphenol	OH	H	H	sec-Butyl	n.d.	n.d.	n.d.
35	H	4-Cyclopentylphenol	OH	H	H	Cyclopenytl	0.09 ± 0.04	n.d.	0.13 ± 0.02
36	H	4-Cyclohexylphenol	OH	H	H	Cyclohexyl	n.d.	n.d.	0.08 ± 0.05
37	H	5-Hydroxyindan	OH	H	H		n.d.	n.d.	n.d.
38	H	5,6,7,8-Tetrahydro-2-naphtol	OH	H	H		n.d.	n.d.	n.d.

*Categories according to Figure 5 in the main text, n.d. = not detected, n.m.= not measured

Catalytic center of ancestral enzymes

A B

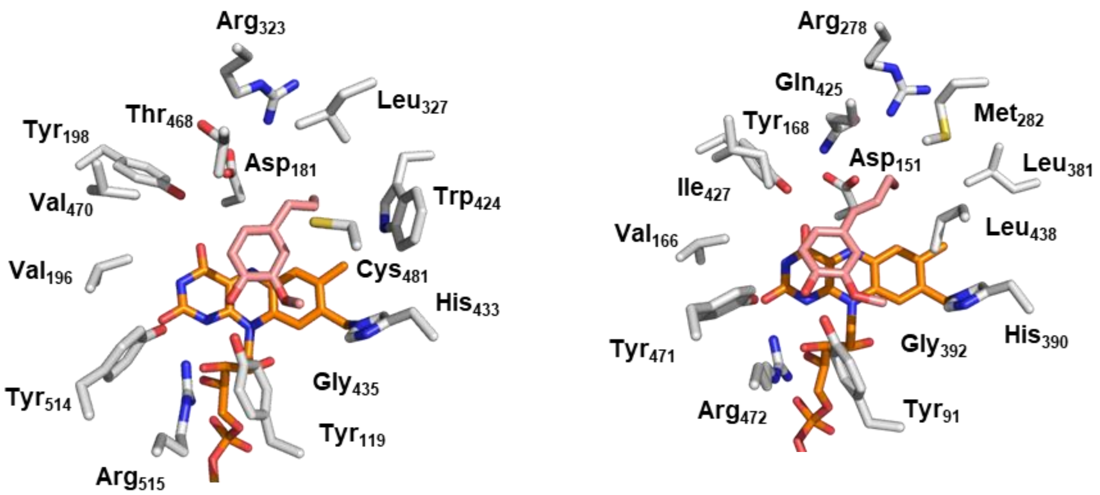

Figure S18. A: Catalytic center of the homology model from AncVAO docked with the substrate eugenol. The model was generated based on the crystal structure of *Ps*VAO (PDB: 2VAO) and *Rj*EUGO (PDB: 5FXP). The FAD cofactor is shown in orange and the docked substrate eugenol is depicted in light red. **B:** Catalytic center from the crystal structure of *Rj*EUGO (PDB: 5FXE) containing coniferyl alcohol which is the conversion product from eugenol. The binding mode in both enzymes is very similar. The FAD cofactor is shown in orange and coniferyl alcohol is depicted in light red.

A B

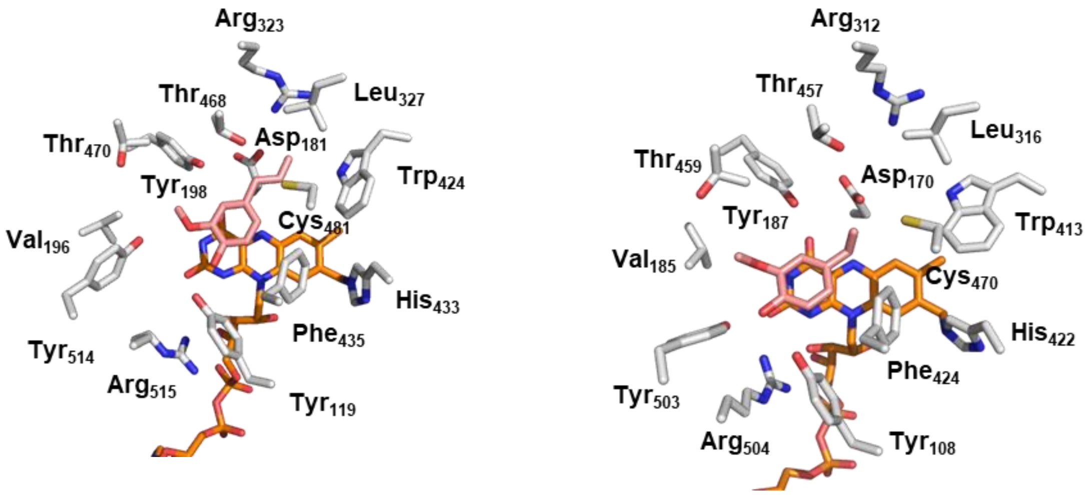

Figure S19. A: Catalytic center of the homology model from Anc*Ps*VAO. The model was generated based on the crystal structure of *Ps*VAO (PDB: 2VAO). The FAD cofactor is shown in orange and the docked substrate eugenol is depicted in light red. **B:** Catalytic center from the crystal structure of *Ps*VAO (PDB: 2VAO) containing bound ethyl phenol. The FAD cofactor is shown in orange and ethyl phenol is depicted in light red.

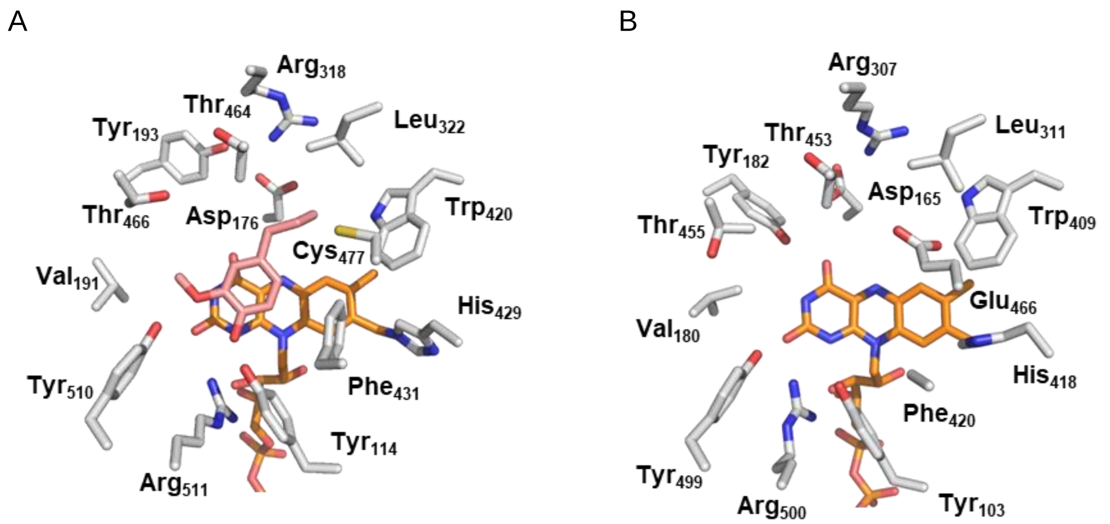

Figure S20. A: Catalytic center of the homology model from Anc*Dc*VAO. The model was generated based on the crystal structure of *Ps*VAO (PDB: 2VAO). The FAD cofactor is shown in orange and the docked substrate eugenol is depicted in light red. **B:** Catalytic center of the homology model from *Dc*VAO. The FAD cofactor is shown in orange.

Protein sequence

>Vanillyl alcohol oxidase [*Diplodia corticola*] codon optimized sequence

ATGAGTCTGTCGAGACCGCTGGCCCTACCCCCCGGCGTATCCGAGTCGACCTTCCATCAGTT-
TATAGAGAGAATAACAAAGACCACGCCTGACGTCACCATCGTCGAAAGCAAAGACCAATTTCCTGACGGAGGCTATCACGAAC
CGCCGAGCTCACATGACCCTTTCTATGTGCAGGACAAAGACCATTTTATTGCGTCCGCATTT-
GTGTGTCCTCGCAGTGTCGATGAAGTTCAAGATATCGTCAGAGCTGCGGGCGACTTCGTCATTCCCCTCTGGCCAACAAGTCT
TGGGCGAAATTTGGGATACGGTGGTGCTGCGCCAAGGGTGCGAGGATCAGTTGTGTTGAA-
TTTGGGTAAACATATGAACCGTATCTTGGAGGTCAATGTGGAGGGCGCGTACGCGCTTGTTGAACCCGGGGTCACATTCGAGG
CGCTACATAATTATCTCGTAGAGCACAATCTTCGTGATAAACTCTGGCTGGATGTCCCG-
GATTTGGGTGGTGGGTCTGTTCTAGGTAACACCCTAGAACGCGGGGTGGGCTATACTCCGTACGGCGATCATTTCATGATGCA
TAGCGGAATGGAAGTGGTATTGGCGAACGGTGAATTGCTGCGCACA-
GGCATGGGCGCTCTGCCTGCCCCAAAAAGTGAGACCGAAGGGGTGACGAAATTACACGAGGAACCTGGTAATAAATGTTGGCA
ACTCTTTCCTTACGGATTTGGTCCATATAATGATGGTTTGTTTAGCCAGTCAAACT-
TAGGTATTGTGACAAAACTTGGCTTGGGATTAATGCCAAATCCAGGAGGGTATCAGTCATATCTGATTACCATCCCAGGCGAA
GAAGATCTGCATAAAGCCGTTGAAATTATTCGGCCATTGCGGTTGAATAACGTAATTCAGAA-
TGTGCCTACTTTACGATATGTGCTTTTAGATGCCGGAATGATGGGACATAAGTCTGATTATACGAGTTCAACTTCACCAATTG
ATGAAGCAACTACGGATGCAATCGCTAAAAAACTTAATCTTGGCCGTTGGAATTTTTATG-
GAGCCCTGTATGGACCACCTGAAATCCGCACCGCCATGTGGAAGCTGATTCATGCTGCTTTTAGTGCTATTCCTGGCGCTCGA
TTTTTTTTTCCTGATGATGTTGCTGATCCTCGTGCCGTACTTCATAATCGTCACAA-
TGTACTTCAAGGTATTCCAAGTTATGAAGAATTACGCTGGGTAGATTGGGTACCAAATGGCGCTCATGTTGCCTTTTCTCCAA
TTTCGCGTATTTCTGGAGCTGATGCAGCACGTCAAGTTGATTTAGCAA-
AGCGTCGCTGTCGTGATGCAGGCTTTGATTATATGGGTACTTTTACAGTAGGCATGCGTGAAATGCATCATATTGTAGAAATT
GTTTTTGATCGTACTGATGCAGGTCAACGTAAACGTGCAGAAGCATTAGTTCGTGGTT-
TAGTTGATGAATGCGCAGCATTAGGTTGGGGTGAATATCGTACACATTTAGCATTTATGGATCAAATTGCAGGTACATATGGT
TGGGGTGGTGGTGCATTAGGTCGTTTTAATGAACGTGTTAAAGAAGCATTAGATCCAA-
AAGGTATTTTAGCACCAGGTAAGTCTGGTGTTTGGGGTTCTCGTTTTGGTGAATTAAAAGGTAAATTATAG

>Vanillyl alcohol oxidase [*Diplodia corticola*] translated protein

MGHHHHHHHHHHSSGHIEGRHMSLSRPLALPPGVSESTFHQFIERITKTTPDVTI-
VESKDQFPDGGYHEPPSSHDPFYVQDKDHFIASAFVCPRSVDEVQDIVRAAGDFVIPLWPTSLGRNLGYGGAAPRVRGSVVLN
LGKHMNRILEVNVEGAYALVEPGVTFEALHNYL-
VEHNLRDKLWLDVPDLGGGSVLGNTLERGVGYTPYGDHFMMHSGMEVVLANGELLRTGMGALPAPKSETEGVTKLHEEPGNKC
WQLFPYGFGPYNDGLFSQSNLGIVTKLGLGLMPNPGGYQSYLITIPGEEDLHKAVEIIR-
PLRLNNVIQNVPTLRYVLLDAGMMGHKSDYTSSTSPIDEATTDAIAKKLNLGRWNFYGALYGPPEIRTAMWKLIHAAFSAIPG
ARFFFPDDVADPRAVLHNRHNVLQGIPSYEELRWVDWVPNGAHVAFSPISRISGADAARQVD-
LAKRRCRDAGFDYMGTFTVGMREMHHIVEIVFDRTDAGQRKRAEALVRGLVDECAALGWGEYRTHLAFMDQIAGTYGWGGGAL
GRFNERVKEALDPKGILAPGKSGVWGSRFGELKGKL

Primer

Table S4: Primer used for Gibson assembly

Name	Sequence
*Dc*VAO_for	CAGCAGCGGCCATATCGAAGGTCGTCATATGAGTCTGTCGAGACCGCTGG
*Dc*VAO_rev	AGCCGGGATCCGATCTACTATAATTTACCTTTTAATTCACCAAAACGAGA
AncVAO_for	AGCAGCGGCCATATCGAAGGTCGTCATATGTCCAGTACGAATAATGTTCC
AncVAO_rev	CAGCTTCCTTTCGGGCTTTGTTAGCAGCCGGATCCTCACAACTCCCACCC
Anc*Dc*VAO_for	CAGCAGCGGCCATATCGAAGGTCGTCATATGAGTTCCACATCCAATGTTC
Anc*Dc*VAO_rev	AGCTTCCTTTCGGGCTTTGTTAGCAGCCGGATCCTCAGGCACCTGGTAAC
Anc*Ps*VAO_for	AGCAGCGGCCATATCGAAGGTCGTCATATGTCTCCAACTAATTCTGTTCC
Anc*Ps*VAO_rev	TCGGGCTTTGTTAGCAGCCGGATCC CTAAAGTTTCCACTCTCTCTTATTG

Table S5: Mutagenesis primer used to introduce E466X mutations into the *Dc*VAO gene by quick change mutagenesis. The mutated codon is highlighted in red.

Name	Sequence
*Dc*VAO_E466C_for	CATATTGTATGCATTGTTTTTGATCGTACTGATGCAGGT
*Dc*VAO_E466C_rev	TCAAAAACAATGCATACAATATGATGCATTTCACGCA
*Dc*VAO_E466L_for	GTGAAATGCATCATATTGTACTGATTGTTTTTGATCGTACTGATGCAGG
*Dc*VAO_E466L_rev	CCTGCATCAGTACGATCAAAAACAATCAGTACAATATGATGCATTTCAC

References

1. Hill, A. V. (1910) A new Mathematical Treatment of Changes of Ionic Concentration in Muscle and Nerve Under the Action of Electric Currents, with a Theory as to their Mode of Excitation. *The Journal of Physiology* **40**, 190–224 10.1113/jphysiol.1910.sp001366 PMID 16993004

2. J. B. S. Haldane, Ed. (1930) *Enzymes,* 3rd Ed.

3. LiCata, V. J., and Allewell, N. M. (1997) Is Substrate Inhibition a Consequence of Allostery in Aspartate Transcarbamylase? *Biophysical Chemistry* **64**, 225–234 10.1016/S0301-4622(96)02204-1 PMID 9127947
